# Mucopenetrative Lipid–Polymer nanoparticles show Potent Anti-Inflammatory activity in a human Lung-on-Chip model

**DOI:** 10.1016/j.ijpharm.2026.126688

**Published:** 2026-02-19

**Authors:** Kalindu D.C. Perera, Alexandra K. Vasta, Jyothi U. Menon

**Affiliations:** a Department of Biomedical and Pharmaceutical Sciences, College of Pharmacy, University of Rhode Island, Kingston RI 02881, USA; b Department of Chemical, Biomolecular, and Materials Engineering, College of Engineering, University of Rhode Island, Kingston RI 02881, USA; c Department of Biomedical Engineering, College of Engineering, Texas A&M University, College Station TX 77843, USA

**Keywords:** Mucus, Mucopenetration, Anti-inflammatory, COPD, Cystic fibrosis, Nanoparticles, Organ-on-chip

## Abstract

Airway mucus presents a significant barrier to inhaled drug delivery, particularly for nanoparticle-based interventions, with this barrier exacerbated in chronic respiratory diseases (CRDs) due to hyperviscous secretions and persistent inflammation. In this study, a dual-functional lipid-polymer hybrid nanoparticle was developed to combine rapid mucolysis with sustained anti-inflammatory activity, and its performance was evaluated using both conventional *in vitro* assays and a physiologically relevant lung-on-a-chip model. Dipalmitoylphosphatidylcholine (DPPC)–coated PLGA nanoparticles (hydrodynamic diameter 378.1 ± 23.0 nm; 58–61 wt% lipid; ζ ≈ +3 mV) encapsulated N-acetylcysteine (NAC) within the lipid shell for rapid release and all-trans retinoic acid (ATRA) within the core for sustained delivery. NAC exhibited a burst release of 44.2–52.5% within 6 h and significantly reduced the viscosity of cystic fibrosis–mimetic mucus, enabling a 26.5-fold higher penetration across a ~ 0.6 mm mucus plug compared to NAC-free controls. The formulation was well tolerated by pulmonary epithelial and fibroblast cells and demonstrated high cellular uptake driven by the DPPC coating. To assess efficacy under physiologically relevant airway conditions, a human lung-on-a-chip model incorporating air–liquid interface, flow, and cyclic stretch was employed. In this model, repeated dosing of NAC + ATRA nanoparticles resulted in a 2.6-fold reduction in IL-6 and a 2.3-fold reduction in IL-8 levels compared to diseased controls at 72 h, outperforming NAC-free nanoparticles at early timepoints and maintaining suppression over 9 days. These findings demonstrate the therapeutic promise of dual-functional mucopenetrative nanoparticles and establish the utility of lung disease-on-chip platforms for evaluating inhaled nanotherapeutics under physiologically relevant conditions.

## 1. Introduction

Chronic respiratory diseases (CRDs) have become the third-leading cause of disease-related mortality worldwide as of 2019, with the Centres for Disease Control of both China and the United States confirming the same trend in 2023 (Ahmad et al., 2024; Lin et al., 2023; Momtazmanesh, 2023). CRDs encompass a range of chronic diseases affecting the respiratory system, including both restrictive and obstructive conditions: chronic obstructive pulmonary disorder (COPD), interstitial lung disease (most commonly, idiopathic pulmonary fibrosis/IPF), asthma, pneumoconiosis, cystic fibrosis (CF) and more (Momtazmanesh, 2023). Together, CRDs were estimated to be responsible for 4.0 million global deaths in 2019, with a prevalence of 454.6 million (Momtazmanesh, 2023). COPD was by far the largest contributor to the former, with 3.3 million deaths worldwide, while asthma was the leading contributor to the latter figure, with 262.4 million cases prevalent (Momtazmanesh, 2023). Driven by smoking, occupational exposure and air pollution among others, CRDs remain a persistent burden on global healthcare resources, with high mortality in low-income nations and high prevalence in high-income nations (Momtazmanesh, 2023). Prevalence is expected to grow as the global population grows and ages, with the global cost of treating COPD alone projected to reach between $4- and 7 trillion in the 2020–2050 period (Chen et al., 2023; Momtazmanesh, 2023).

CRDs present with a variety of different pathophysiological features: common amongst these is persistent inflammation of lung tissue, particularly the airways (Lee et al., 2021). While the primary end-result of unresolved inflammation is airway obstruction and alveolar degradation, the associated tissue remodelling has an impact on other systems within the respiratory environment as well. One of these is the function of the goblet cells and submucosal glands, whose production of mucus is significantly altered during the progression of CRDs. In the healthy lung, airway mucus consists of approximately 97.5% water, 1.1% globular proteins, 0.9% salt and 0.5% mucins (along with small quantities of DNA, lipids, and assorted cellular material) (Abrami et al., 2024; Hill et al., 2022). The mucins, of which MUC5AC and MUC5B are the most prominent (MUC2, MUC8, and MUC19 being present in smaller quantities), are large glycoproteins with characteristic serine and threonine-rich regions (Abrami et al., 2024; Hill et al., 2022). Mucins are capable of interacting both chemically and physically with each other and with other mucus components to form a mesh-like structure that acts as an adhesive barrier between the airway lumen and epithelial mucosa, with pore sizes of between 10 and 1000 nm (Lock et al., 2018; Pangeni et al., 2023). In addition, healthy human airway mucus is known to have a bulk viscosity of 12–15 × 10^3^ centipoise (cP) (Ponomarchuk et al., 2020). By comparison, the viscosity of mucus in a CF-affected lung can be 14–110 × 10^3^ cP (Ponomarchuk et al., 2020). This increase in viscosity can be observed in a wide array of CRDs and can be caused by two main factors: changes in mucin expression, or mucus dehydration (leading to hyperconcentration) (Abrami et al., 2024; Hill et al., 2022). Other factors can include goblet cell hyperplasia and submucosal gland hypertrophy (Abrami et al., 2024). Aside from the dehydration observed in CF mucus, most of these changes have been linked to underlying inflammation in multiple studies (Abrami et al., 2024). CRD-affected mucus tends to be more adhesive (preventing facile mucociliary clearance combined with the increased viscosity) and have smaller pore sizes (thereby trapping more inhaled pathogens and irritants, further contributing to the underlying inflammation) (Abrami et al., 2024). This increase in viscosity is at times accompanied by an increase in the amount of mucus produced by the airway epithelium, further congesting the small, distal airways and serving as a breeding ground for assorted pathogens (Abrami et al., 2024).

Airway mucus is thus a clear physiological barrier to inhaled drug delivery- doubly so when the lungs are affected by a CRD. As such, mucus-interacting nanoparticles (NPs) are not a new phenomenon in the field of nanomedicine. Several types of mucoadhesive NPs have been formulated and described in literature, seeking to circumvent a common problem posed by mucus to some NPs: short retention time (El-Sherbiny et al., 2015; Jalal et al., 2023; Yan and Sha, 2023). Such NPs have exploited a host of properties that allow them to bind to the mucin chains, through careful selection of materials to generate the desired chemical or charge-dependent interaction with mucins (Butnarasu et al., 2022; Jalal et al., 2023; Rencber et al., 2016; Silva et al., 2017). Mucopenetrative NPs have not been as common- these NPs seek to penetrate the mucus mesh rapidly, utilizing muco-inert coatings/materials and/or tuned sizes and surface charges to wholly avoid interactions with the mucins. The underlying logic is one that calls for NPs to sidestep any mucus interactions that would result in their rapid clearance either through mucociliary action or expectoration and reach the underlying mucosa within a short timeframe (Lai et al., 2009). In this scenario, several properties are of interest: NPs are expected to have a near-neutral surface charge to avoid charge repulsion or attraction (preventing mesh penetration), surface chemistry that precludes chemical interactions with mucus components, and a size that is within the 100–300 nm range to further enhance penetration through the mesh pores (Alp and Aydogan, 2020; Lai et al., 2009; Perera et al., 2015). Some examples of work in this space include those such as by Popov and co-workers, who showed that coating otherwise mucoadhesive nanoparticles with selected partially hydrolyzed poly(vinyl alcohol) yielded particles that rapidly diffused through cervicovaginal mucus, overturning the conventional view of PVA as intrinsically mucoadhesive (Popov et al., 2016). Others engineered pegylated poly(aspartamide)-polylactide nanoparticles with tunable PEG content, identifying near-neutral formulations that efficiently penetrated cystic fibrosis-mimetic artificial sputum and were proposed for pulmonary delivery of ibuprofen (Craparo et al., 2016). Ways and colleagues systematically grafted short non-ionic polymers such as PEG, poly(2-hydroxyethyl acrylate), poly(2-ethyl-2-oxazoline), or poly(N-vinylpyrrolidone) onto chitosan, generating nanoparticle formulations whose diffusion through model mucus was greatly enhanced compared with unmodified chitosan (Ways et al., 2022). Maniyamgama and co-workers designed ionizable lipid-incorporated liquid lipid nanoparticles whose soft liquid core and PEGylated, near-neutral surface (achieved by tuning the ionizable/cationic lipid ratio to match nasal mucosal pH) confer muco-penetrating properties for intranasal mRNA delivery across airway mucus (Maniyamgama et al., 2025). The inclusion of active mucolytic agents within nanoformulations to further enhance their ability to move rapidly through the mucus barrier is another recent thrust of research in this area: Pereira de Sousa and colleagues described a poly(acrylic acid) nanoparticle with surface-conjugated mucolytic enzymes for intestinal applications (Pereira de Sousa et al., 2015). Their work found bromelain-conjugated NPs to be more effective at mucus permeation compared to papain-conjugated counterparts. In addition to size, charge, and surface hydrophilicity, recent studies have begun to explore particle geometry and mechanical properties as additional design parameters for transport through mucus and other biological hydrogels. For example, tadpole-like polymer/lipid Janus nanoparticles with asymmetric geometry and semielastic stiffness show enhanced penetration through nasal mucus compared with conventional spherical, rigid particles, and broader reviews of hydrogel transport highlight nanoparticle elasticity as a key determinant of diffusion through mucus, extracellular matrix, and related gels (Okmen Altas et al., 2024; Sodimanage and Schneider, 2025).

The complex interplay between mucus rheology, airway inflammation, and drug transport underscores the need for both therapeutic strategies and evaluation systems that reflect the physiological environment of diseased airways. Despite substantial progress in the development of mucopenetrative nanoparticles, most evaluations have been confined to static *in vitro* systems, typically involving 2D cell cultures or mucus penetration assays. Such models lack critical physiological features of the lung, including air–liquid interface, mechanical stretching, and directional flow, all of which influence nanoparticle transport and therapeutic performance (Chen et al., 2021; Ingber, 2022; Park et al., 2024). Organ-on-chip platforms that mimic these features have emerged as promising tools to bridge this gap, yet nanoparticle systems designed for mucus penetration and anti-inflammatory action have not previously been evaluated in such models.

In this study, a dual-functional lipid-polymer hybrid nanoparticle was formulated by incorporating a mucolytic and an anti-inflammatory agent (N-acetylcysteine (NAC) and all-trans retinoic acid (ATRA), respectively) into a single inhalable platform, and its performance was assessed using both conventional *in vitro* approaches and on a lung-on-a-chip model that reproduces key features of the human airway. We hypothesized that spatial compartmentalization of these agents (with NAC in a dipalmitoylphosphatidylcholine (DPPC) lipid shell for rapid mucus modulation and ATRA in a PLGA core for sustained anti-inflammatory release) would enable sequential barrier-conditioning and therapeutic delivery in diseased airway mucus. Accordingly, DPPC-coated PLGA nanoparticles were prepared and evaluated for mucus-thinning and longer-term anti-inflammatory effects. Thus, the novelty of this work lies in combining a dual-compartment, sequential-function nanoparticle design with evaluation in a human airway organ-on-chip under air-liquid interface, flow, and cyclic stretch, enabling assessment of mucus barrier modulation and anti-inflammatory outcomes in a physiologically dynamic setting.

## 2. Experimental

### 2.1. Materials

All material were purchased from Sigma Aldrich (St. Louis MO, USA) unless otherwise stated. Acetonitrile, ammonium thiocyanate and bovine serum albumin (BSA) were purchased from Alfa Aesar and both DAPI and MTT reagent were obtained from EMD Millipore (Burlington MA, USA). All-trans retinoic acid (ATRA), the bicinchoninic acid (BCA) assay kit, iron chloride hexahydrate and N-acetylcysteine (NAC) were purchased from Thermo Scientific (Waltham MA, USA), as were Gibco penicillin–streptomycin and trypsin-EDTA (0.25%). Anhydrous ethanol was obtained from Pharmco (Mississauga ON, Canada). Coumarin-6 and diethylenetriaminepentaacetic acid (DTPA) were purchased from Acros Organics (Geel, Belgium). Dipalmitoylphosphatidylcholine (DPPC), egg yolk enrichment, Matrigel and Tween-20 were purchased from Avanti Polar Lipids (Alabaster AL, USA), Remel (Lenexa KS, USA), Corning (Corning NY, USA) and Bio-Rad (Hercules CA, USA), respectively. Recombinant human IL-1β was generously provided by Fujifilm Irvine Scientific (Irvine CA, USA). Spectra/Por dialysis tubing (12,000–14,000 kDa M_W_ cutoff) was purchased from Repligen (Waltham MA, USA), while 300-mesh carbon film copper transmission electron microscopy (TEM) grids and UranyLess were sourced from Electron Microscopy Sciences (Hatfield PA, USA). GenClone Dulbecco’s Modified Eagle Medium (high glucose with L-glutamine and sodium pyruvate) was purchased from Genesee Scientific (Morrisville, NC), while fetal bovine serum was purchased from Innovative Bioscience (Benicia CA, USA). Artificial mucus was purchased from Biochemazone (Leduc AB, Canada), while ELISA MAX Deluxe Human IL-6 and −8 ELISA kits were purchased from BioLegend (San Diego CA, USA). A549 pulmonary epithelial cells and MRC-5 pulmonary fibroblasts were sourced from ATCC (Manassas VA, USA). Lung-chips and associated equipment and consumables were purchased from Emulate (Boston MA, USA).

### 2.2. Nanoparticle synthesis

The synthesis of lipid-polymer hybrid nanoparticles (NPs) was carried out following protocols previously published by our group (Gonsalves et al., 2023; Perera et al., 2025). To synthesize the core polymer NP, a 20 mg/mL solution of carboxyl-terminated poly(lactic-coglycolic) acid (resomer RG 503H, 50:50 lactide:glycolide ratio, M_w_ 24,000–38,000; PLGA) in chloroform was added dropwise to a 5% w/v solution of poly(vinyl alcohol) (PVA; M_W_ 13,000–23,000, 87–89% hydrolyzed) in ultrapure water under constant stirring. The resulting oil-in-water suspension was subjected to pulsed probe sonication using a FB120 Sonic Dismembrator (Fisher Scientific, Waltham MA, USA) for 5 min. This emulsion was allowed to stir overnight to allow for solvent evaporation. Particles were collected following ultracentrifugation at 25,000 rpm, 10°C in an Optima L-100 XP ultracentrifuge (Beckman Coulter, Brea CA, USA) in two 30-minute steps, followed by freezing and lyophilization to obtain the final NPs.

PLGA with a molecular weight of 24–38 kDa was selected for the synthesis of the core NP as it lies within a widely used range for pulmonary nanoparticle formulations, offering a balance between particle stability and sustained drug release. Within this molecular weight window, nanoparticle size and morphology are primarily governed by formulation and processing parameters rather than polymer molecular weight alone (Mittal et al., 2007; Reis et al., 2025; Stromberg et al., 2021).

Two modifications were made to the above protocol when synthesizing fluorescent- or drug loaded NPs for experiments described herein. For the former (C6-PLGA NPs), coumarin-6 was dissolved directly in the PLGA-containing oil phase at 0.8 mg/mL prior to dropwise addition into the water phase. Similarly, ATRA was dissolved directly in the oil phase at polymer:drug mass ratios of 20:1 and 10:1 during optimization. Final synthesis of drug-loaded particles (ATRA-PLGA) was carried out at a polymer:drug mass ratio of 10:1.

Coating of the pre-formed PLGA NPs to produce the final hybrid (DPPC-PLGA) NPs was carried out by means of thin-film hydration. A solution of DPPC and cholesterol in chloroform (at 2.5- and 0.088 mg/mL respectively) was dried into a thin film under reduced pressure at 50°C by means of a R-100 rotary evaporator (Buchi, Flawil, Switzerland), maintaining a ratio of 1 mL of lipid solution per mg of NPs. This film was subjected to hydration at 50°C for 1 h with a 0.66 mg/mL suspension of the PLGA NPs in ultrapure water. The resultant suspension was subjected to pulsed probe sonication for 7 min and dialyzed against ultrapure water for 5 h at 4°C to remove excess, non-coating lipid. Dry particles were collected by means of lyophilization and stored at −20°C until further use. NAC was encapsulated within the lipid layer (DPPC-NAC-PLGA) by direct dissolution of the drug in the rehydration medium at a mass ratio of 1:50 drug:PLGA NPs prior to rehydration. This approach was based on exploratory work done in-house as well as prior liposome-based work done by others (Emre Yalcin et al., 2021; Gonsalves et al., 2023; Sadarani et al., 2019; Xu et al., 2012).

All downstream analyses were conducted on particles post-lyophilization, after reconstituting them in the appropriate solution.

### 2.3. Physical characterization

To determine the basic physical characteristics of the NPs, they were first evaluated for hydrodynamic size and surface charge using dynamic light scattering (DLS) and zeta potentiometry *via* a Zetasizer Nano ZS (Malvern Panalytical, Malvern, UK). Brightfield TEM was carried out using a JEM-2100 TEM (JEOL, Tokyo, Japan); samples were prepared by placing a drop of dilute NP suspension on a copper TEM grid followed by UranyLess negative staining and allowing air-drying overnight. Attenuated total reflectance Fourier-transform infrared (ATR FT-IR) spectroscopy was performed on pre-desiccated samples using an IRTracer-100 spectrometer (Shimadzu, Kyoto, Japan). IR transmittance spectra were acquired in the 400–4000 cm^−1^ range at a 2 cm^−1^ spectral resolution and 24 scans per sample. Thermogravimetric analysis (TGA) was carried out using a TA Instruments TGA 55 (Waters, Milford MA, USA) instrument, over a range of 25 to 500°C at a 10°C ramp within a nitrogen atmosphere. Determination of onset temperature was performed manually on OriginLab. A Stewart assay was also carried out to determine the efficiency of the lipid coating process and the composition of the final NP formulation, adapting a previously published protocol (Stewart, 1980). Briefly, DPPC-PLGA and DPPC-NAC-PLGA were dispersed in chloroform at 1 mg/mL and shaken at room temperature for 10 mins. Stewart reagent was prepared by the dissolution of ammonium thiocyanate and iron chloride hexahydrate as directed on the protocol. Following shaking, the NP samples were incubated for 30 mins in the dark with the Stewart reagent, following which the lower chloroform phase was subjected to measurement on a Genesys 50 UV–vis spectrophotometer (ThermoScientific, Waltham MA, USA) at λ = 470 nm. Quantification of lipids in samples was carried out by comparison to a standard curve made using neat DPPC in chloroform processed as above.

### 2.4. Assessment of particle stability in suspension

To confirm the in-suspension stability of the NPs, DPPC-PLGA NPs were suspended in ultrapure (MilliQ) water, PBS (pH 7.4) and simulated lung fluid (Gamble’s fluid, prepared as outlined by Marques *et al*. (Marques et al., 2011)), and incubated at 37°C on an orbital shaker. DLS and zeta potentiometry was carried out on aliquots drawn from these suspensions every 24 h for a period of 7 days.

### 2.5. Evaluation of drug encapsulation and release kinetics

To determine the encapsulation efficiency (EE%; see Eq. (1)) and loading (see Eq. (2)) of drug-loaded NPs, the lyophilized NPs were dispersed in an organic solvent. For NAC, 5 mg/mL of DPPC-NAC-PLGA NPs were subjected to 6 h of agitation in 100% ethanol on a rotary shaker. For ATRA, 5 mg/mL of DPPC-ATRA-PLGA NPs were subjected to the same in a 1:1 mix of ethanol and acetonitrile. The samples were centrifuged, and the supernatants subjected to UV–vis absorbance spectrophotometry for quantification (λ_NAC_ = 208 nm; λ_ATRA_ = 350 nm).

(1) $$EE\%=\frac{\text{total}\;\text{encapsulated}\;\text{drug}\left(mg\right)}{\text{total}\;\text{drug}\;\text{added}\;\text{during}\;\text{synthesis}\left(mg\right)}\times 100$$

(2) $$\text{Loading}=\frac{\text{total}\;\text{encapsulated}\;\text{drug}\left(\mu g\right)}{\text{total}\;\text{mass}\;\text{of}\;\text{NPs}\left(mg\right)}$$

Release kinetics of both drugs were assessed in PBS at two pH values, 6.5 (pH of inflamed airway) and 7.4 (standard pH for *in vitro* evaluation). 2 mL of a 4 mg/mL suspension of DPPC-NAC-PLGA or DPPC-ATRA-PLGA NPs in either medium was placed within dialysis bags (12–14 kDa M_W_CO), sealed and placed in 5 mL of the same medium within 50 mL centrifuge tubes protected from light. Sink conditions were maintained throughout this study. This setup was incubated at 37°C on an orbital shaker and 500 μL aliquots drawn from it at predetermined timepoints, with fresh medium replacing it. Aliquots were stored at −20°C until quantification was carried out *via* UV–vis spectrophotometry as above. Release was expressed as a cumulative percentage of encapsulated drug.

To characterize the underlying release mechanisms, cumulative release data were subsequently fitted to established kinetic models using nonlinear regression in GraphPad Prism (v9.0.0). Burst + Higuchi, Burst + First-order, and Weibull models were evaluated using user-defined equations (see Table S1). Model parameters were estimated by least-squares fitting of cumulative release (%) versus time (h). For ATRA, cumulative release plateaued below 100%, and A_max_ was fixed to the mean release at the final time point to stabilize parameter estimation. For NAC, which approached complete release, A_max_ was left unconstrained. Goodness of fit was evaluated using the coefficient of determination (R^2^) and residual analysis to compare model performance.

### 2.6. In vitro assays

All cells were grown in Dulbecco’s Modified Eagle Medium (DMEM) supplemented with 10% fetal bovine serum (FBS) and 1% penicillin/streptomycin and maintained in standard culture conditions (37°C, 5.0% CO_2_) unless otherwise indicated. All cell detachment steps were performed using 0.5% Trypsin-EDTA.

The nanoparticle concentrations used here (1–3 mg/mL) were selected as an upper-bound range to approximate local levels that can arise within the small airway surface liquid volume (thin ~ 7 μm airway surface liquid (Song et al., 2009; Tarran et al., 2006)) following deposition of concentrated inhaled suspensions, and are comparable to concentrations employed in other nanoparticle exposure studies with A549 and related lung cell models (Altube et al., 2022; De Matteis et al., 2022; Zhang et al., 2018).

#### 2.6.1. Cytocompatibility studies

To assess cytocompatibility of the developed NP formulation, A549 lung epithelial cells and MRC-5 lung fibroblasts were first seeded at a density of 5000 cells/well on 96-well plates. These were treated with the appropriate concentration of treatment agent: IL-1β was assessed at concentrations of 0–20 ng/mL at 24, 48 and 72 h timepoints, while DPPC-NAC-ATRA-PLGA NPs were assessed at 0–2 mg/mL over 24–48 h. MTT assays were carried out with 2 h incubation once the MTT reagent was added, per the manufacturer’s instructions. Absorbance measurements were carried out on a Synergy H1 spectrophotometer (BioTek, Winooski VT, USA) at λ = 570/630 nm.

#### 2.6.2. Optimization of epithelial inflammation model

To establish the optimal conditions for inducing inflammation *in vitro*, A549 cells were seeded at a density of 10,000 cells/well on 96-well plates. Upon cell attachment, treatment with 0–20 ng/mL of IL-1β was carried out over 24-, 48- or 72 h. Media supernatant was collected and stored at −20°C until subjected to quantification of IL-6 and −8 (indicators of airway inflammation) *via* ELISA.

#### 2.6.3. Uptake by pulmonary epithelial cells

To assess cellular uptake of the formulation by the airway epithelium, A549 cells were used as a model and seeded at a density of 10,000 cells/well on 96-well plates. Based on results of 2.6.2, cells were exposed to 5 ng/mL IL-1β for 48 h to induce inflammation (alongside non-inflamed controls). Serum starvation was carried out to synchronize cell cycles using serum-free DMEM (+/− 5 ng/mL IL-1β as appropriate) for 2 h, following which C6-containing NP groups (PLGA and DPPC-PLGA) were introduced in complete media (+/− 5 ng/mL IL-1β as appropriate) at concentrations of 0–2 mg/mL. The treated cells were incubated at standard conditions for 2 h, following which cells were washed with PBS and 250 μL/well 1% Triton X-100 in PBS was placed in all wells. Well floors were gently scratched, and the plates were left overnight at 4°C to allow for cell lysis. Plates were subjected to UV–vis absorbance spectrophotometry at λ = 550 nm, alongside standards of dye-encapsulating NPs made in cell culture medium (separate standards were made for DPPC-coated and non-coated NPs to fully account for subtle optical- and dye-content differences). BCA assay was used to quantify total protein content in each well. Results were reported as micrograms of NP uptake per microgram of cellular protein. Representative images of NP uptake by cells were obtained using an EVOS M5000 microscope (Invitrogen, Waltham MA, USA). Cells were treated as outlined above (up to NP addition), fixed using 4% paraformaldehyde in PBS for 10 mins. and counterstained with DAPI prior to imaging.

### 2.7. Evaluation of mucolytic activity

The studies described below were performed using a previously published *in vitro* model of diseased human airway mucus developed/described by Yang *et al*., unless otherwise stated (Yang et al., 2010). Briefly, mucin (from porcine stomach, type II; 5 mg/mL), egg yolk enrichment (5 μL/mL), DNA (10 mg/mL), RPMI-1640 amino acids 20 μL/mL), DTPA (6 μg/mL), NaCl (5 mg/mL) and KCl (2.2 mg/mL) were dispersed in DNAse-free water in quantities as specified in the published protocol and mixed for 2 h at room temperature prior to use.

#### 2.7.1. Mucus plug penetration assays

To evaluate the penetrative ability of the NPs, an approximately 625 μm thick layer of mucus was deposited on cell culture inserts (12-well plate; 12 mm diameter, 3 μm pore size) and shaken vigorously on an orbital shaker for 10 mins. to ensure uniform distribution. 40 μL of a 25 mg/mL fluorescent NP suspension (in DNAse-free water) of DPPC-C6-PLGA or DPPC-NAC-C6-PLGA was placed at the centre of each mucus plug. DPPC-C6-PLGA was plated on its own and with EE (DPPC-NAC-PLGA)-adjusted free NAC (1.5 μg). 500 μL of DNAse-free water was placed in the recipient compartment, and the plate incubated at 37°C in the dark. 100 μL of the contents of the recipient compartment was removed at predetermined timepoints (1, 3, 6 and 24 h) and stored at −20°C until needed, with fresh, pre-warmed DNAse-free water being added to the compartment to replace the volume sampled. Sampled aliquots were subjected to UV–vis spectrophotometry to quantify the NPs that crossed the mucus plug and culture insert membrane at each time point (λ = 550 nm).

#### 2.7.2. Bulk rheology

To assess the effects of the developed NPs on the rheological properties of mucus, a Discovery HR30 rheometer (TA Instruments, New Castle DE, USA) with 20 mm cone geometry was used. Samples of synthetic mucus were subjected to a strain sweep (10 rad s^−1^, 0.01–100% strain) to determine their linear viscoelastic regions (LVRs). Frequency- and flow sweeps within the LVR were carried out on the mucus samples with appropriate treatment groups at 0-, 3- and 6 h. Treatments groups were as follows: DNAse-free water-only (control), free NAC (106.3 μg/mL, the total released NAC from 3 mg of NPs in 6 h), DPPC-PLGA, DPPC-ATRA-PLGA and DPPC-NAC-ATRA-PLGA (all at 3 mg/mL). Frequency sweeps were run at 1.0% strain amplitude across frequencies of 100- to 0.1 rad/s, while flow sweeps were carried out at a shear rate of 100.0- to 1 × 10^−3^ s^−1^. All data analysis was performed on TA Instruments’ TRIOS software (v5.1.1).

### 2.8. Evaluation of anti-inflammatory activity in vitro

#### 2.8.1. Preliminary evaluation of anti-inflammatory action in 2D culture models

To confirm the basic anti-inflammatory properties of NP components and the final formulation, A549 cells were seeded at a density of 10,000 cells/well on 96-well plates, then treated with 5 ng/mL of IL-1β for 48 h (alongside non-treated control). Cells were treated with NAC (58.3 μg/mL), ATRA (5.1 μg/mL) and DPPC-NAC-ATRA-PLGA NPs (1 mg/mL). The concentration of NAC and ATRA were adjusted to match the cumulative concentration of each released by the NPs at 72 h. Media supernatant was collected at timepoints of 24-, 48- and 72 h, and stored at −20°C until subjected to quantification of IL-6 and −8 *via* ELISA.

#### 2.8.2. Evaluation of anti-inflammatory action in physiologically relevant organ-chip models

To more accurately model the therapeutic response of inflamed airways to the NPs developed herein, Emulate Inc.’s lung-on-a-chip platform was utilized in tandem with ELISA-based quantification of key pro-inflammatory cytokines. Organ chips were seeded with A549 cells at a density of 1 × 10^6^ cells/mL as directed on vendor protocols. Cells were left to incubate under standard flow conditions (30 μL/hr) and air–liquid culture initiated with mechanical stretching (5% stretch at 20 Hz) for 7 days until mucus production occurred (Plebani et al., 2022). In all but healthy controls, IL-1β (5 ng/mL)-containing media was introduced through the chips’ bottom channels to induce inflammation for 1 week, and cells in the top channel were supplemented with synthetic mucus from Biochemazone. NP treatments were administered through the top channel in mucus-containing media at a concentration of 2 mg/mL, by high flow (200–300 μL/hr) for 1 h followed by cessation of flow. Bottom channel flow was maintained at 30 μL/hr throughout the experiment. Samples of chip effluent media were collected and stored (at −20°C) at 3-, 6- and 9 days post-NP administration to assess the effects of the NPs at longer timepoints, and levels of IL-6 and −8 were quantified using ELISA.

### 2.9. Graphing, statistical analysis and image processing

All graphing (with the exception of FTIR and TGA data) was carried out on GraphPad Prism (v9.0.0; GraphPad Software Inc., USA); FTIR and TGA data (as well as rheological trend data in the Supplementary Information) were plotted on OriginLab (v10.1.0.178; OriginLab, USA). All statistical analysis was performed on Prism. ImageJ was utilized (v1.54 g; NIH, USA) to perform scale bar additions for micrographs (Schneider et al., 2012).

## 3. Results and Discussion

### 3.1. Particle sizing and basic characterization

Airway mucus presents a significant barrier for the efficacy of inhaled therapeutics, particularly those in nano- or microencapsulated forms that can adhere to various components of the mucus, including *via* H-bonding, electrostatic interactions, hydrophobic interactions, disulfide linkages or simply by steric exclusion (Zheng et al., 2025). Predictably, this is worsened when mucus hypersecretion and/or hyperviscosity occurs. Various strategies of circumventing these issues have been explored over the years, with the majority centred around the coating of particles with inert coatings such as poly(ethylene glycol) or its derivatives (Habibi et al., 2023; Poinard et al., 2019; Sato et al., 2025; Zhang et al., 2023; Zheng et al., 2025).

By way of an alternative strategy, we sought to design and evaluate a novel formulation intended for inhalation delivery, based on a lipid-polymer hybrid nanoparticle design. The DPPC-based outer lipid coating was hypothesized to impart two key properties to the formulation: rapid release of a mucolytic (NAC) to enable unimpeded movement of the particles through respiratory mucus, and an inherently non-mucoadherent surface to further enhance mucus penetration. Several groups have demonstrated that DPPC–partly by virtue of its zwitterionic nature–is an effective muco-inert material (Costabile et al., 2024; Pangeni et al., 2023). In addition, being a primary component of lung surfactant, the DPPC coating imparts a protective effect on intact NPs moving through airway mucus, minimizing clearance by macrophages within the mucus. This is an effect our group has demonstrated in a recent publication, where we showed that lung surfactant-coated nanoparticles were capable of evading macrophage uptake to a considerable degree (Gonsalves et al., 2023). The PLGA-based inner core was envisioned to act as a firm, supportive substructure for the coating, primarily functioning as a means by which an anti-inflammatory agent (ATRA) could be delivered in a sustained manner to the inflamed epithelium underlying the mucus. Together, this was expected to be able to penetrate quicker through respiratory mucus and lead to a more significant reduction in inflammation than formulations lacking either functionality. Two model drugs were chosen for inclusion within the delivery formulation herein: N-acetylcysteine is a US Food and Drug Administration (USFDA)-approved mucolytic drug found in many over-the-counter formulations (U.S. Food and Drug Administration, 2022); ATRA (an endogenous retinoid metabolite) is a similarly USFDA-approved drug, for a variety of anti-inflammatory and anti-neoplastic applications (McCarthy, 1995). ATRA was chosen as a model anti-inflammatory agent due to its well-documented immunomodulatory effects and established roles in reparative epithelial differentiation and barrier regulation under inflammatory conditions (Callaghan et al., 2020; Snyder et al., 2020; Yamada and Kanda, 2019). Although clinical data for ATRA in chronic respiratory diseases remain limited, prior *in vitro* studies—including PLGA-based nanoparticle formulations—have demonstrated its ability to attenuate key pro-inflammatory cytokines such as IL-6 and IL-8 in airway-relevant inflammatory models (Payne et al., 2019). Moreover, its hydrophobicity, instability and narrow route of exerting its anti-inflammatory effects make it a relevant candidate for encapsulation-based delivery approaches, allowing evaluation of sustained release and barrier-mediated transport without relying on broadly immunosuppressive corticosteroids (Dolin et al., 2023; Rhen and Cidlowski, 2005). Finally, PLGA too has received USFDA approval, while DPPC (a constituent of lecithin) is designated Generally Recognized As Safe (GRAS) and is found in a multitude of food and drug products (Fayyaz et al., 2024; Lim et al., 2022).

**Fig. 1.**
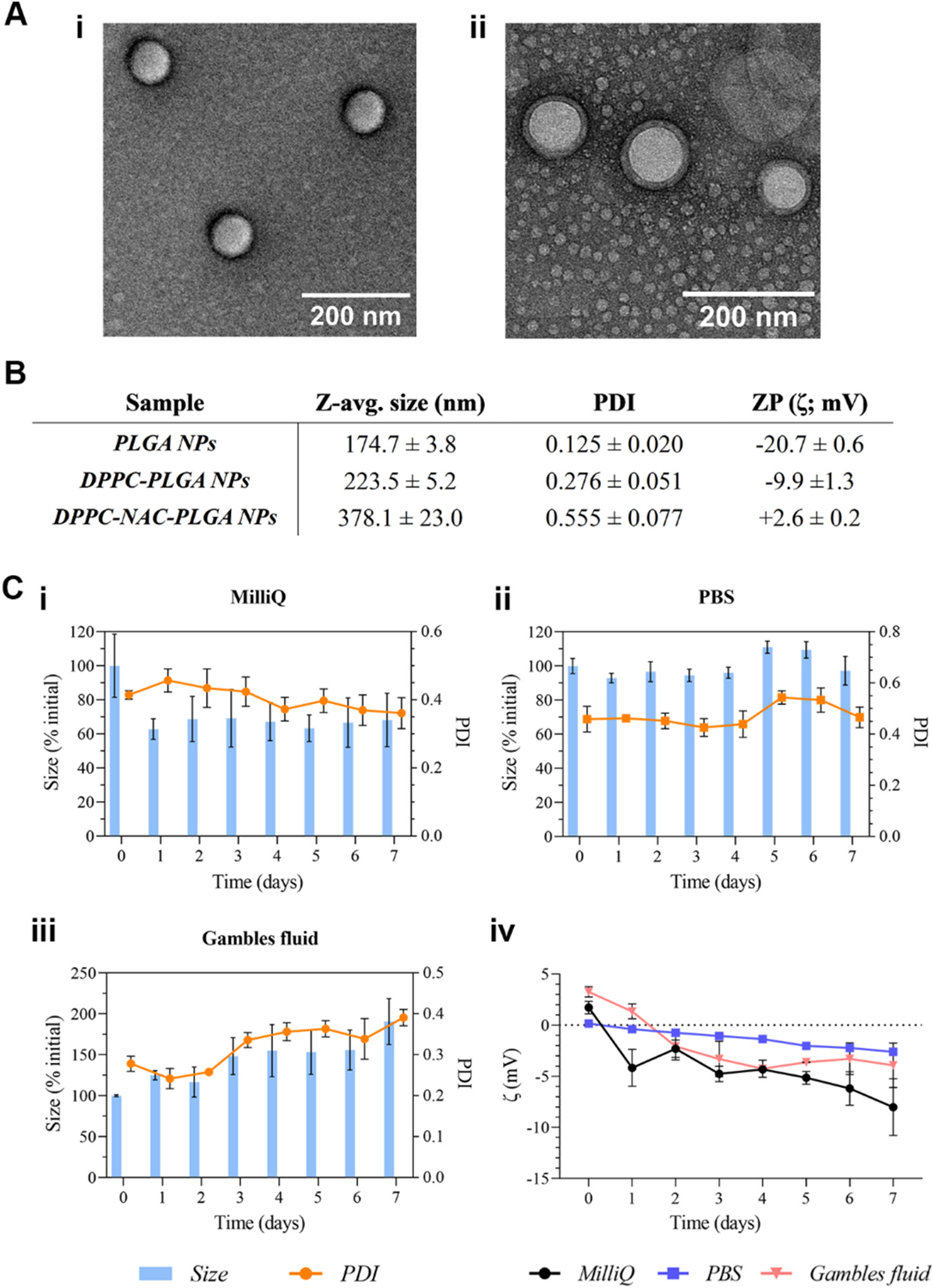
Summary data of basic characterization of the formulation. A) TEM images of the NPs in their **i)** bare (PLGA) and **ii)** coated forms (DPPC-NAC-ATRA-PLGA; lipid coating clearly visible); **B)** DLS/zeta potential (ZP) data of NPs at various stages of synthesis; and **C)** results of a 7-day stability study, examining particles’ size and polydispersity in **i)** ultrapure water (MilliQ), **ii)** PBS, pH 7.4 and **iii)** simulated lung fluid, and **iv)** examining their ZP in all media (all at *n* = 3).

To this end, we synthesized nanoparticles of a size compatible with inhalation delivery (Fig. 1A). Increased polydispersity was observed once coated (Fig. 1B), which was to be expected given variations in individual particles’ coating density after the thin film hydration process. The inclusion of the mucolytic led to further increases in particle diameter, indicating the successful incorporation of the drug in the lipid layer and possible changes to the arrangement of lipids within the coating. This increase in size was reflected in the polydispersity index as well, which increased to 0.555. ATRA-encapsulating NPs caused significant interference to measurements *via* DLS. Due to the NPs’ core–-shell architecture, ATRA is expected to release from the PLGA core into the lipid shell and subsequently into the surrounding bulk medium. The rapid or ‘burst’ ATRA release from the lipid shell initially on particle resuspension likely interfered with DLS measurements. Thus, NP dispersity and size were visualized instead using electron microscopy only. NPs experienced an approximate halving of zeta potential (ZP) post-coating, indicating successful coating of the zwitterionic DPPC lipid. This is consistent with values observed in literature, which all report a significantly low value of ζ for liposomes or other formulations involving neat DPPC as compared to standard PLGA NPs (Chibowski and Szczés, 2016; Liu et al., 2015). It is of note, however, that the encapsulation of NAC within the lipid layer causes a further change in ζ, causing it to increase to approximately + 3 mV.

Fig. 1C presents the results of a 7-day stability study of the DPPC-PLGA formulation performed in various media, with Gamble’s fluid being an established model of the interstitial fluid in the deep lung, which these particles may encounter following inhalation and uptake. The data herein indicates that the DPPC-coated particles may initially undergo partial rearrangement or sloughing off of the lipid shell in electrolyte-free media. This hypothesis is supported by the change in ZP of these NPs, where ultrapure water (MilliQ) appeared to cause a rapid decline in ZP within the first 24 h of the study (indicating possible exposure of the PLGA core, which has a more negative ZP- see Fig. 1B), while the other media elicited a slower decline in the same period. The NPs were fairly stable in PBS, indicating that no lipid shell loss or change in properties is expected in the nebulization medium. Although particle size increase was observed in Gamble’s fluid (consistent with reports that the high ionic strength and divalent cations in Gamble’s solution promote media-dependent agglomeration and restructuring of lipid/protein coronas, leading to gradual increases in hydrodynamic size in simulated lung fluids (Hachenberger et al., 2023; Innes et al., 2021; MacCuspie et al., 2011; Sommer, 2021)), this was not statistically significant after day 1. In addition, although suspensions with near-neutral ZPs can, in principle, be more susceptible to aggregation, the combination of a zwitterionic, highly hydrated DPPC corona and gradual exposure of the more negatively-charged PLGA core in physiologic media was sufficient to preserve colloidal integrity over the 7-day window studied: only modest increases in size and PDI were observed and no visible precipitation occurred. This last observation is of particular relevance to the *in vivo* delivery of these NPs: it suggests that freshly reconstituted DPPC-PLGA nanosuspensions possess sufficient short-term colloidal stability to be compatible with delivery in suspension form, provided they are briefly resuspended immediately prior to use.

Due to their high diffusive mobility, an aerodynamic diameter in the 100–500 nm range has been deemed optimal for nanoparticle deposition in the deep lung once inhaled: our group has been among many that have demonstrated this in the past (Cojocaru et al., 2024; El-Sherbiny et al., 2015; Menon et al., 2014; Praphawatvet et al., 2020; Wan et al., 2023). These patterns of deposition and retention would almost certainly be affected by the properties of excess and/or hyperviscous airway mucus. A study by Porro and colleagues, for instance, found large numbers of particles as small as 100 nm being trapped in the mucus from the airways of patients afflicted by cystic fibrosis (Porro et al., 2010). The mesh size of CRD mucus is significantly smaller than that of healthy airway mucus, with both CF and COPD mucus demonstrated to trap particles in the 100–500 nm range particularly well (Chisholm et al., 2019; Duncan et al., 2016). This initial trapping may enable high NP retention *in vivo*, followed by a hypothesized rapid disengagement from the mucus mesh *via* NAC action. Our goal was to balance two competing needs, the first being a size large enough to enable deposition/trapping in the smaller airways where inflammation in CRDs is most capable of causing impactful remodelling and blockages due to excessive mucus production or viscosity. The second was for the NPs to be small enough to enable facile uptake by target cells. While a generally accepted upper limit for efficient cell uptake of nanoparticles is 200 nm, it is important to note that this comes with several caveats, one of which is surface modification (Hoshyar et al., 2016; Mazumdar et al., 2021). In the case of the current formulation, the lipid coating’s partial shielding of PLGA’s surface charges becomes doubly important. Firstly, a near-neutral zeta potential has been established to be optimal for reduced interaction with the mucus mesh (Bandi et al., 2020; Cobarrubia et al., 2021; Lai et al., 2009; Liu et al., 2021; Lock et al., 2018; Yuan et al., 2023). While particles with a negative charge tend to be repelled (primarily) by the negatively charged mucin-associated glycans and their carboxyl and sulfate groups, those with a positive charge interact strongly with these and other anionic groups in mucus (Bandi et al., 2020; Cobarrubia et al., 2021; Lai et al., 2009; Liu et al., 2021; Lock et al., 2018; Yuan et al., 2023). In either case, such properties make for poor mucopenetration by particles. Conversely, particles with a weak or near-neutral surface charge such as that of our final formulation undergo minimal interaction with the mucus mesh and are thus ideal for applications requiring mucopenetration. Secondly, a positive charge on NPs helps with increased internalization at a target cell membrane given its negative charge, although high positive charges can lead to cytotoxicity (Fröhlich, 2012; Lin et al., 2014). This was particularly well-demonstrated by Xiao and colleagues, who showed that while NPs with >+10 mV charge outdid more modestly positively charged counterparts in cell uptake, those with a ζ approximately the same as our formulation (+3 mV) performed significantly better at being cytocompatible (Xiao et al., 2011). Taken together, the above-discussed factors and the data presented in Fig. 1 point to our formulation being of a size and charge well-suited for deep-lung deposition and retention, cell uptake, and mucopenetration.

### 3.2. Confirmation of coating and physicochemical characterization

**Fig. 2.**
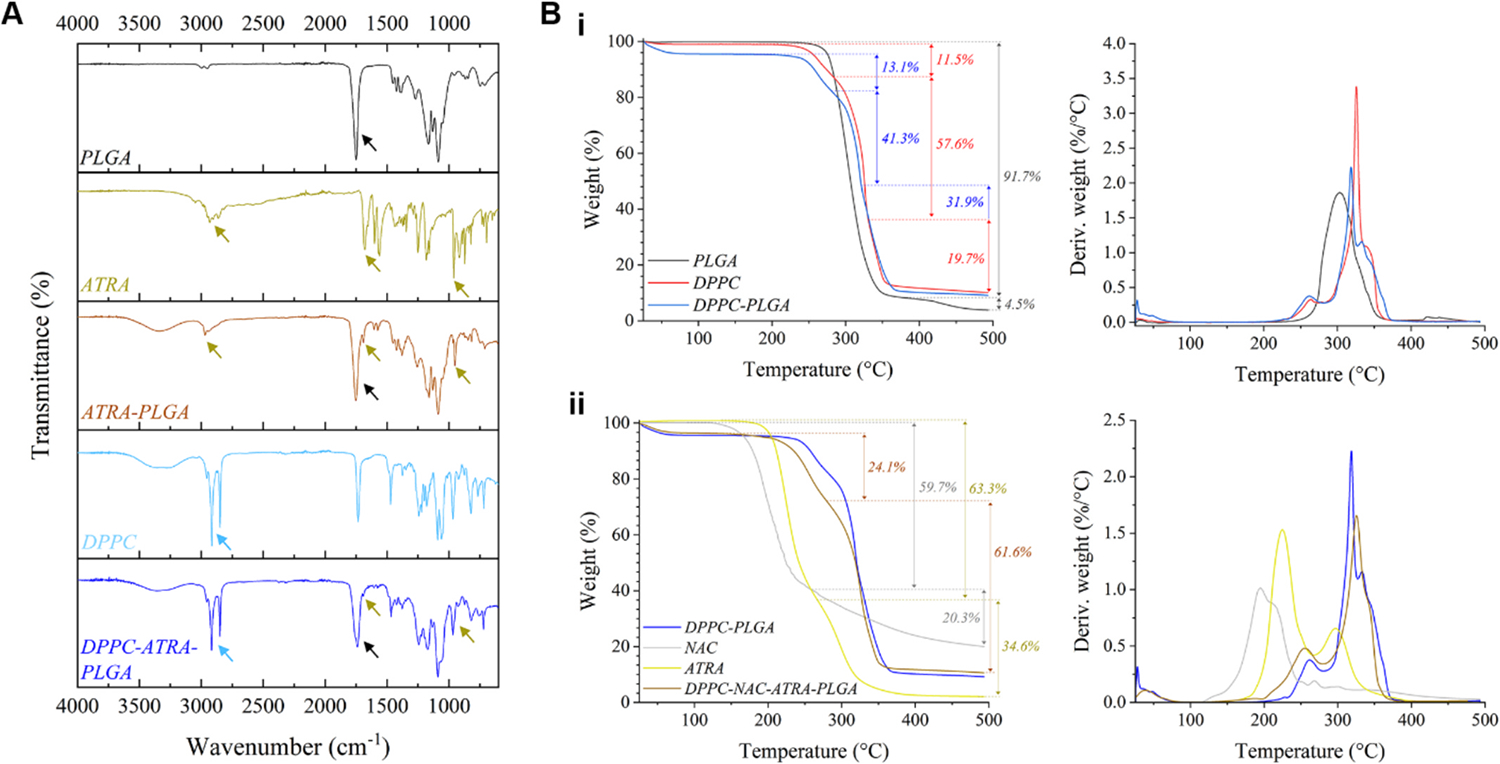
Physicochemical characterization of the formulation. **A)** presents FT-IR spectra that confirm encapsulation of ATRA within the PLGA core, and the successful application of the DPPC lipid coating on the core NP. **B)** presents TGA data for **i)** evaluating and confirming lipid coating of PLGA NPs and **ii)** encapsulation of both NAC and ATRA within the final formulation. See Table 1 for quantitative summary of TGA data.

Confirmation of NP coating by DPPC was achieved using three methods. Firstly, a Stewart assay-based quantification of DPPC-PLGA and DPPC-NAC-PLGA NPs revealed that the particles consisted of 58–61% DPPC by weight, which is in alignment with previous work involving lipid-polymer hybrid NPs published by our group (Gonsalves et al., 2023). FT-IR analysis was also carried out for this purpose (see Fig. 2A) and showed clear incorporation of DPPC into the PLGA nanoparticle by way of the presence of the characteristic twin peaks of the methylene stretching vibrations at 2914 cm^−1^ (*v*_as_(–CH_2_)) and 2849 cm^−1^ (*v*_s_(–CH_2_)) (Disalvo et al., 2022) in both the DPPC and DPPC-ATRA-PLGA spectra (light blue arrows). FT-IR also provided confirmation of the successful incorporation of ATRA into the PLGA matrix, with the presence of a system of peaks near 3000 cm^−1^ (usually attributed to *v* (C–H) and *v*(O–H) vibrations), the *v*(C=O) vibration at 1680 cm^−1^ and the δ(C=C) vibration at 961 cm^−1^ in both the neat ATRA and ATRA-PLGA spectra (gold arrows) (Khoshbakht et al., 2020; Kim et al., 2016; Shakiba et al., 2017). While the first peak system is obscured by the stronger DPPC methylene stretch peaks in the DPPC-ATRA-PLGA spectrum, the other two are visible in that spectrum (although at a lower intensity, with the *trans*-substituted vinyl stretch at 961 cm^−1^ becoming a shoulder of a peak produced by the DPPC). The characteristic *v*(C=O) vibration of PLGA(Carmagnola et al., 2020) at 1750 cm^−1^ is preserved through all PLGA-containing spectra (indicated by arrows in black), confirming its presence in all such samples; spectra of NAC-inclusive NPs (see Fig. S1 of Supplementary Information) showed no characteristic peaks of NAC in the nanoformulation- its presence was confirmed by other means as outlined below.

Thermogravimetric analysis (TGA) was also utilized to confirm both coating (Fig. 2B–i) and drug encapsulation (Fig. 2B-ii). DPPC coating of PLGA yielded a product with a thermal decomposition profile very similar to neat DPPC, both having undergone a three-step decomposition. PLGA’s presence in the lipid-polymer hybrid NPs was indicated by the DPPC-PLGA’s marked resistance to decomposition at step 2 (see Table 1), which was likely a function of the thermally-more stable PLGA core. Together, these served to confirm inclusion of DPPC into the PLGA sample. Incorporation of NAC and ATRA into the coated NPs caused a further drop in thermal stability (as indicated by onset and peak decomposition temperatures), likely owing to the more delicate thermal decomposition profiles of the two small molecules. This was particularly noticeable in the onset temperature of the first decomposition step of the final formulation, which decreased by 17.6°C, very likely due to NAC (with its low onset temperature of 171.5°C). The presence of NAC can also be confirmed by comparison of residual masses: neat NAC has a much higher residual mass (20.0%) than that of any other component of the final formulation and is thus likely the reason for the increased residual mass of the final formulation (10.6%) relative to that of drug-free coated NPs (9.1%).

In summary, analysis *via* Stewart assay, FTIR and TGA confirmed the presence of DPPC within the formulation, as well as the successful encapsulation of both the mucolytic NAC and anti-inflammatory ATRA within the formulation’s structure.

### 3.3. Drug encapsulation and release kinetics

**Fig. 3.**
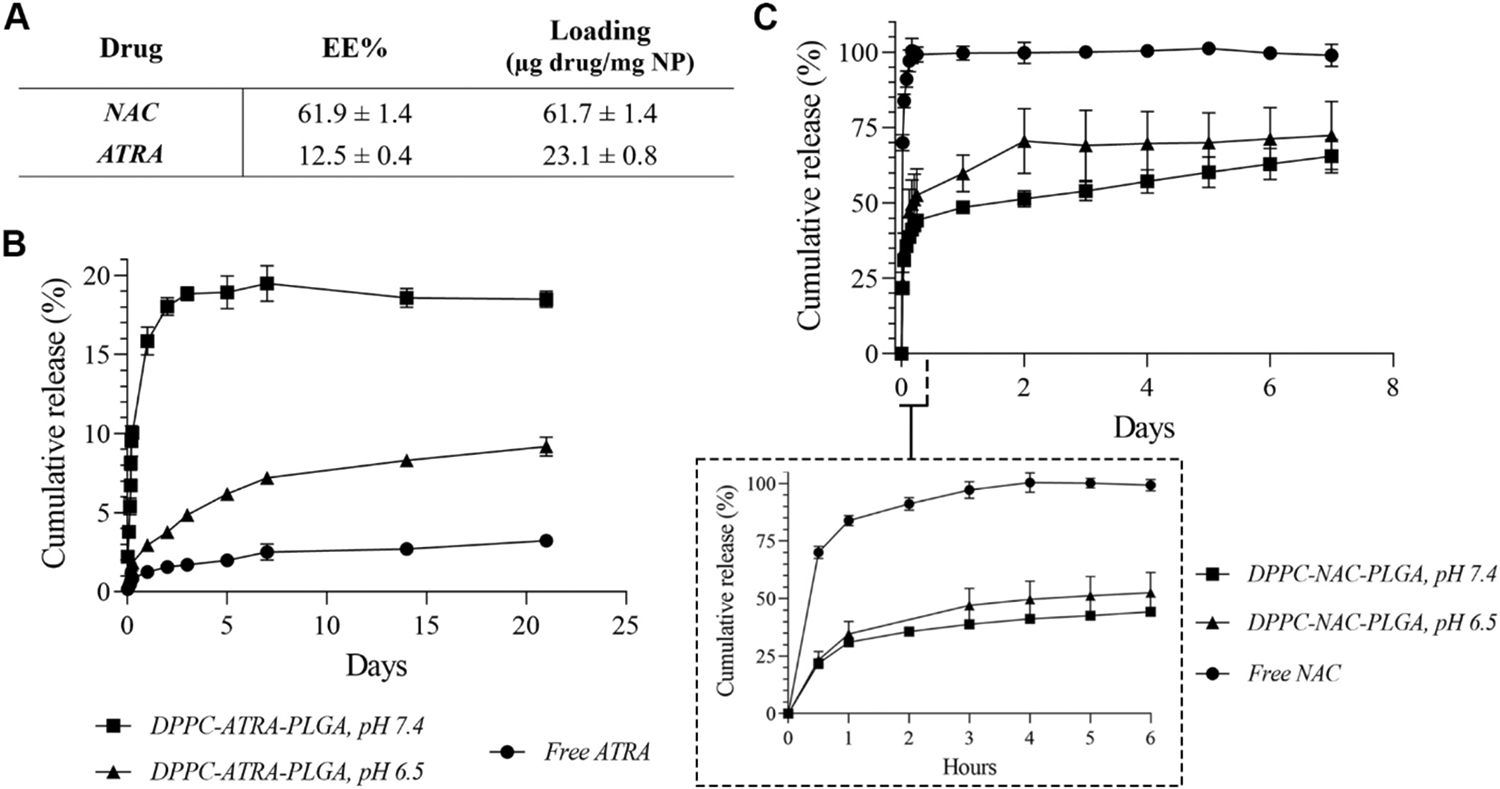
Assessment of the encapsulation in- and release of drugs from the final NP formulation. **A)** summarizes encapsulation and loading data for both NAC and ATRA; **B)** shows the release pattern of ATRA from both the free and nanoencapsulated forms; **C)** presents the release pattern of NAC in both free and lipid-encapsulated forms. All release evaluation performed at *n* = 3. See Table S2 for quantification of remaining drug in each matrix at each tested time point.

Quantitative data relating to encapsulation and loading of both NAC and ATRA is presented in Fig. 3A. Release of each of the drugs was carried out in PBS separately, at two pH values. pH 6.5 was used to replicate the slightly acidic environment of the microbially-infected inflamed airway tissue, while pH 7.4 was used as to replicate the slightly more alkaline pH of generally inflamed airways (as well as the standard assayed pH for drug release) (Zajac et al., 2021). Release of ATRA from DPPC-ATRA-PLGA NPs (Fig. 3B; see Fig. S3 for calibration plots) at pH 7.4 showed an initial burst release of 10% within the first 6 h, followed by a sustained first-order release phase, reaching 19.5% by day 7. This is quite low compared to extant data on retinoic acid/PLGA systems where cumulative release frequently approaches a minimum of 60% (Chen et al., 2018; Jeong et al., 2008; Júnior et al., 2023; Yi et al., 2020), but matches work by Chae and colleagues, who observed sub-20% cumulative release from PLGA matrices over the same period (Chae and Oh, 2019). At the more acidic pH, release was retarded by a significant margin compared to the release at pH 7.4; 1.8% of total encapsulated drug was released in the initial burst release over 6 h. A slower rate of release was then observed until day 21 for a peak release of 9.2% of total encapsulated drug. This slower rate may be beneficial for a drug such as ATRA, protecting it from metabolism (being susceptible to the retinoid-metabolizing CYP26 in particular) and oxidative degradation within the pulmonary environment (Stevison et al., 2017, 2015). Given that ATRA has been shown to be effective at levels on the order of nanograms per millilitre *in vivo* (Mirza et al., 2006), we did not envision this slow release to present a considerable obstacle for therapeutic efficacy. Free ATRA experienced predictably low release (just 3% peak release by day 21) from the dialysis membrane setup used herein, showing that nanoencapsulation of the drug significantly improves the aqueous dispersibility of this poorly-water soluble drug.

The release of NAC from the DPPC coating (Fig. 3C) was performed over a shorter time than that of ATRA, as the initial burst release of a mucolytic from the lipid shell was hypothesized to enable the rapid delivery of the core to the target site. As a result, release was evaluated up to a maximum of 6 h, as this is the longest period these particles are expected to remain uncleared/unabsorbed by cells within the airways. Under these conditions, the particles displayed release kinetics similar to those published on NAC-lipid nanocarrier systems, where approximately 50% of the drug was released within a 6-hour period, and remaining so up to the conclusion of their study at 9–10 h (Hamedinasab et al., 2020; Hamedinasab et al., 2023). Initial burst release within the first 6 h resulted in the release of 52.5- and 44.2% of total encapsulated NAC at pH 6.5 and 7.4 respectively. This early period of release is of importance, as these NPs must be capable of moving rapidly through the mucus mesh, aided by rapid release of large quantities of mucolytic from their lipid shell. This rapid movement helps avoid clearance through expectoration or other means. The results of this release study (particularly the high burst release) appear to indicate that this requirement has been fulfilled, although the release of NAC at the pH of mucus found in the inflamed airway (pH 7.4) was marginally (approximately 8%) lower than that in the more acidic pH. Free NAC, assessed as a control, experienced rapid diffusion out of the dialysis membrane setup, attaining 100% release within 6 h, in keeping with the drug’s high aqueous solubility and dispersibility.

To better understand the mechanisms underlying the observed release profiles, data were fitted to multiple kinetic models as outlined previously, including burst + Higuchi, burst + first-order, and Weibull models (see Fig. S2). NAC release from the DPPC-PLGA nanoparticles was most accurately fitted by the Weibull model at both pH 7.4 (R^2^ = 0.956) and pH 6.5 (R^2^ = 0.891). This indicated a complex, non-Fickian release process likely arising from a combination of rapid diffusion from the lipid layer and a slower release phase associated with NAC located in less accessible regions within or beneath the DPPC coating.

ATRA release from the nanoparticle formulations was best described by the burst + first-order model at both pH 7.4 (R^2^ = 0.9845) and pH 6.5 (R^2^ = 0.9867), with modest initial burst fractions (2.0% and 1.1% at pH 7.4 and 6.5, respectively) followed by sustained first-order release. The markedly lower k_1_ at acidic pH (0.0084 h^−1^ vs. 0.110 h^−1^ at pH 7.4) reflects slower PLGA degradation and reduced diffusivity of ATRA within the polymer matrix under these conditions, in line with the slower cumulative release observed experimentally. Although the DPPC surface coating may contribute to suppression of the initial burst and act as a minor diffusion barrier, the sustained release of ATRA is governed primarily by the PLGA core. Notably, no Weibull fits were required for ATRA, as the conventional burst + first-order model adequately captured the release kinetics across both pH conditions.

Taken together, the results of the release studies show firstly that our final formulation exhibits a rapid (approx. 50% release within 6 h at both tested pH values) release of NAC from the outer DPPC shell, possibly enabling more rapid diffusion of these NPs through CRD-affected airway mucus than a formulation without the mucolytic. Secondly, we show that the release of ATRA from the PLGA core, particularly that at the pH of inflamed airway epithelium, is a low but sustained pattern over a 21-day period, with the potential of establishing a depot effect within inflamed cells through repeated dosing, helping ameliorate chronic CRD-related inflammation.

### 3.4. Cellular interactions in vitro: Cytocompatibility and uptake

**Fig. 4.**
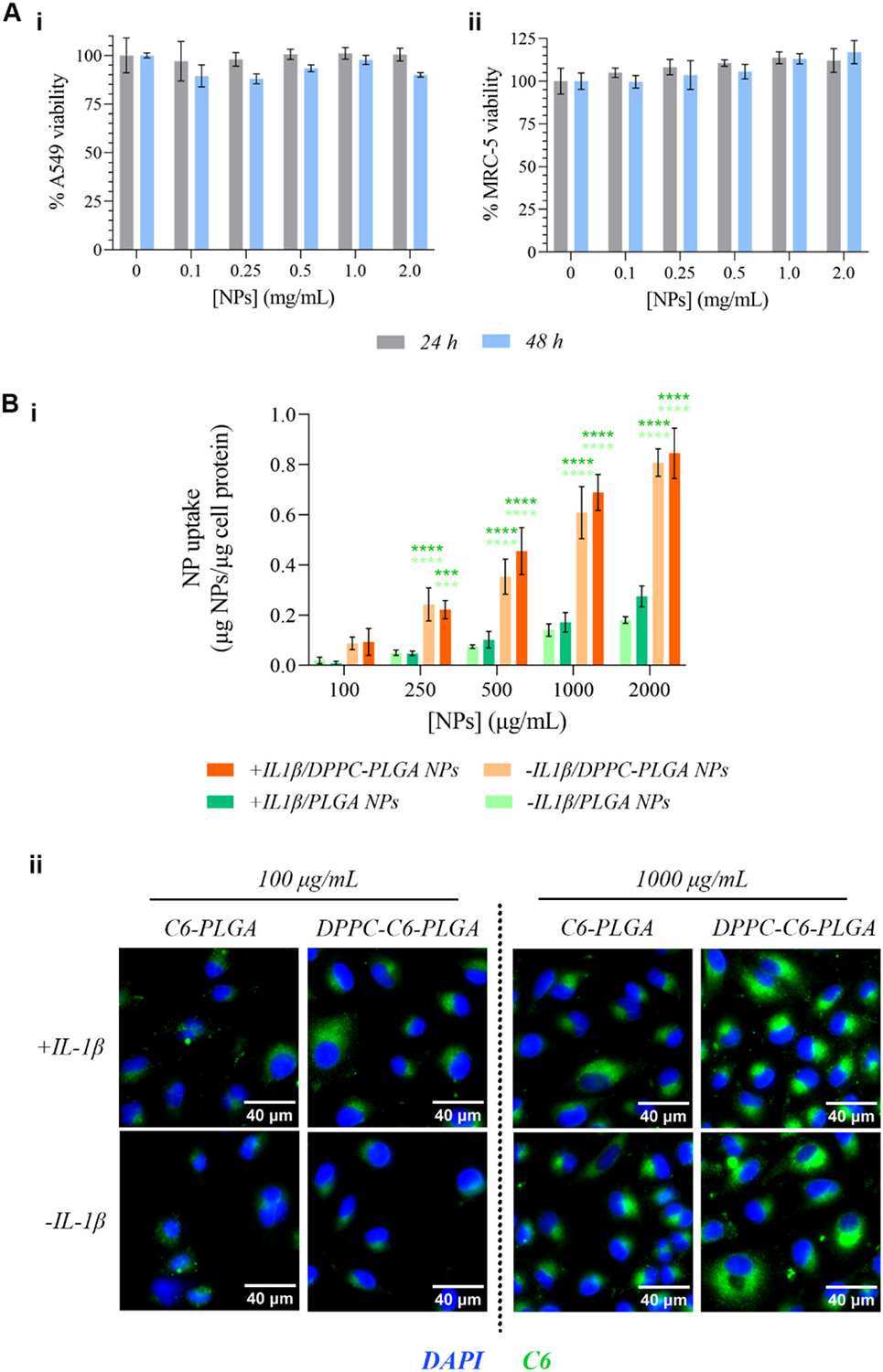
Preliminary evaluation of NP performance *in vitro*, in the form of **A)** cytocompatibility studies (*n* = 4) vs. **i)** A549 pulmonary epithelial cells, and **ii)** MRC-5 pulmonary fibroblasts, and **B)** cell uptake studies, in the form of both **i)** quantitative analysis (*n* = 4) and **ii)** representative qualitative imaging. (*** P ≤ 0.001, **** P ≤ 0.0001).

To act as a preliminary form of toxicological evaluation, our formulation was evaluated for cytocompatibility against two common types of cells that they are likely to encounter *in vivo*: their intended primary target, epithelial cells, and the ultrastructure-forming fibro-blasts. As such, A549 lung epithelial cells were utilized to model the former (Fig. 4B–i), while MRC-5 lung fibroblasts were used to model the latter (Fig. 4A-ii). A549 cells were selected as the epithelial component of the *in vitro* studies herein as it is a well-established surrogate for human type II alveolar epithelium and is widely used to study pulmonary drug delivery and nanoparticle–cell interactions, particularly under ALI conditions (relevant to organ-on-chip work: see Fig. 6) (Lee and Rezaee, 2024; Leibrock et al., 2019). While primary alveolar epithelial cells from CF, asthmatic, or COPD donors would offer additional disease specificity, they are difficult to obtain in sufficient numbers and exhibit substantial donor-to-donor variability and limited expansion potential, which makes them less suitable for initial formulation optimization (Hasan et al., 2018; Hiemstra et al., 2018). These studies with model cells done at 24- and 48 h showed no significant cytotoxicity as > 80% cell viability was observed for both cell lines at both timepoints.

As noted previously, the near-neutral/slightly positive ζ of the DPPC-coated NPs was expected to facilitate higher cell uptake. This trend was noted definitively during a nanoparticle cell uptake study with A549 cells, quantitative analysis of which is presented in Fig. 4B–i, with representative fluorescence micrographs provided in Fig. 4B-ii. C6-encapsulating PLGA NPs with or without the DPPC coating was incubated with healthy (−IL-1β) or inflamed (+IL-1β) epithelial cells for 2 h.

DPPC coating markedly increased cellular uptake of PLGA nanoparticles in both inflamed and non-inflamed epithelial cells (being taken up at approximately 4–5-fold of that experienced by non-coated counterparts at all tested concentrations), whereas uncoated PLGA particles showed only minimal internalization. Given that our DPPC-coated formulations were approximately 300 nm in diameter and near-neutral in zeta potential (C6 encapsulation did not change ZP), this size places them within the regime where macropinocytosis and other clathrin-independent pathways are known to contribute substantially to nanoparticle uptake (Foroozandeh and Aziz, 2018; Means et al., 2022; Rennick et al., 2021). The clear dose-dependency observed at all tested concentrations with no sign of saturation in uptake even at 2.0 mg/mL lends further credence to this.

The C6-loaded DPPC-PLGA nanoparticles used in the cell uptake studies were prepared by the same nanoprecipitation and DPPC-coating procedure as the formulations characterized by DLS (Fig. 1B) and are therefore expected to have hydrodynamic diameters within a similar range (approximately 250–300 nm). Co-encapsulation of NAC and ATRA in DPPC-PLGA typically increases the mean particle diameter toward the upper end of this window (up to ~ 400 nm), although reliable DLS measurements of ATRA-containing particles were not possible under our instrument conditions due to optical interference from ATRA. Particles in the 250–400 nm size regime are generally internalized *via* macropinocytosis and other clathrin-independent pathways, suggesting that modest differences in size between the C6-loaded and NAC/ATRA-loaded formulations may affect the magnitude of uptake but are unlikely to alter the qualitative behavior observed here.

We therefore demonstrate that our formulation is well-tolerated by cells most likely to interact with these NPs within the pulmonary environment and may be taken up by lung epithelial cells at a particularly high level owing to the lipid coating and the properties it imparts to the NP.

### 3.5. Evaluation of mucolytic activity and mucus penetration

N-acetylcysteine’s mucolytic effect is primarily actioned through its ability to hydrolyse disulfide linkages, a property demonstrated both *in vitro* and *in vivo* as early as the 1960 s (Hurst et al., 1967; Sheffner et al., 1964). Mucin chains are known to possess cysteine-rich regions both at their termini as well as in their mid-chain domains, which engage in end-to-end polymerization and inter-chain crosslinking respectively (Aldini et al., 2018), An S_N_2 thiol-disulfide exchange between NAC’s inherent thiol group and the disulfide bonds that form the mucus mesh has been posited to be the most likely route by which these disulfide links are broken, thus reducing the viscosity of mucus found in the airways of CRD-afflicted individuals (Aldini et al., 2018; Sadowska, 2012).

**Fig. 5.**
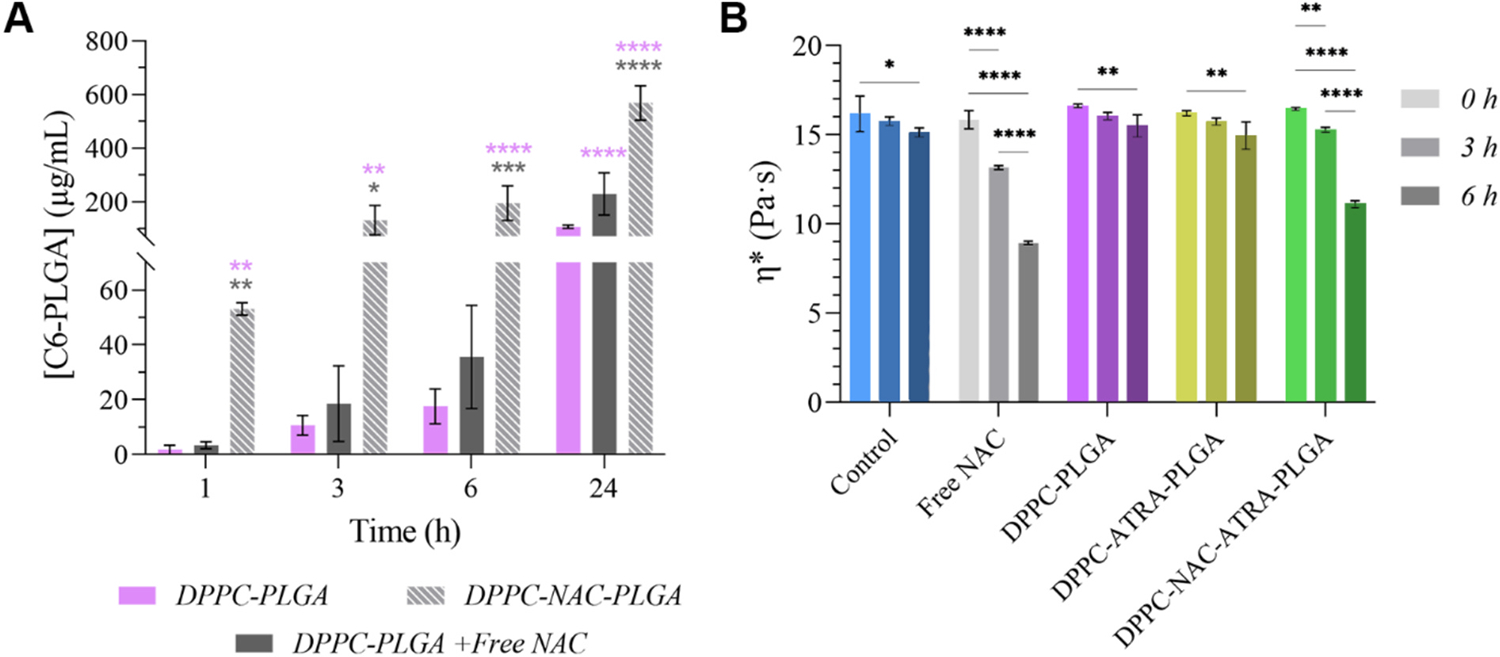
Evaluation of NPs’ mucolytic activity. **A)** summarizes data from a mucus plug diffusion assay (*n* = 3) carried out on a cell culture insert system; **B)** presents results of bulk rheology work done with the final DPPC-NAC formulation and controls (*n* = 3). (* P ≤ 0.05, ** P ≤ 0.01 *** P ≤ 0.001, **** P ≤ 0.0001).

To assess the mucus-thinning properties of the formulation, an initial mucus plug diffusion assay was carried out using an *in vitro* model of artificial mucus that has been well-established in literature (Fig. 5A). First synthesized by Yang and colleagues, the mucus has been designed to replicate the properties of mucus present in the airways of cystic fibrosis (CF) patients (Yang et al., 2010). Its rheological properties–particularly viscosity–were confirmed to fit within the accepted range of viscosity observed in *ex vivo* samples of CF mucus (see Fig. S4) (Puchelle et al., 1985; Radiom et al., 2021). Suspensions of NPs (corresponding to 1 mg of DPPC-PLGA, DPPC-PLGA with free NAC or DPPC-NAC-PLGA) containing C6 were deposited on a 625 μm-thick layer of mucus upon the apical side of a porous cell culture insert. This thickness was used as a reasonable balance between mucus thickness in air ways of patients with COPD (up to 300 μm (Abrami et al., 2024)) and in mucus plugs observed in the same (>1000 μm (Kim et al., 2025)), with similar thicknesses having been employed by others in related experiments modelling the CF airway surface liquid (Worlitzsch et al., 2002).

Quantification of aliquots from the basal compartment drawn at multiple timepoints revealed that NAC-encapsulating NPs outperformed the other two groups by a factor of 16.6–26.5 as early as 1 h after assay initiation. 26.6 μg (translating to 2.7% of administered NPs) of DPPC-NAC-PLGA NPs were found to have migrated fully through the mucus layer and the porous culture insert’s membrane into the acceptor compartment at this time, compared to just 3.4- (0.3%) and 1.8 μg (0.2%) of DPPC-PLGA + free NAC and DPPC-PLGA respectively. Several important trends are thus notable from this data: firstly, the incorporation of NAC into the lipid coating appears to confer a more focused and therapeutically relevant mucolytic effect to the NPs. This is seen in the difference (at all timepoints) in diffused NP content between the encapsulated and non-encapsulated NAC groups. While it is likely that free NAC dispersed throughout the NP suspension allows that group to outdo the NAC-free formulation, its failure to match the nanoencapsulated NAC supports the idea that DPPC-NAC-PLGA NPs were able to exert a highly localized mucolytic effect that allowed individual/clusters of NPs to penetrate through the mucus. Secondly, this assay shows that the burst release of NAC at a timescale of just 1 h (corresponding to approx. 21.4 μg of NAC/mg NPs- see Fig. 3) is sufficient for markedly higher penetration through the mucus mesh. It indicates that the formulation may be capable of rapid movement through viscous CRD airway mucus at short timescales following administration *in vivo*. This also underscores the importance of encapsulation, since non-encapsulated NAC is unable to replicate this rapid effect. Finally, the persistence of the significantly elevated mucolytic effect of nanoencapsulated NAC even at 24 h is heartening, as it provides further support to the importance of such encapsulation.

To confirm the release of NAC and preservation of its mucolytic activity post-encapsulation, the effect of NAC on the bulk rheological properties of the model mucus was also assessed, with results presented in Fig. 5B. Evaluated over 6 h, a DNAse-free water vehicle control and a free NAC positive control (added at an amount matching the total NAC released within 6 h by 3 mg of NPs) were compared to DPPC-PLGA, DPPC-ATRA-PLGA and DPPC-NAC-ATRA-PLGA (all at 3 mg/mL to allow for measurable changes in bulk viscosity on the available instrument). While there was a significant reduction in the complex viscosity (η*) of the mucus over time (as the model mucus breaks down naturally; visible particularly in the negative control), the effect of free NAC and the NAC-encapsulating NPs far outstripped this effect, being highly significant at both non-zero timepoints. Given that the nanoencapsulated NAC requires release from the DPPC shell, encapsulated NAC was expected to experience a slight lag in effect compared to free NAC. It is nevertheless of note that the released NAC can cause bulk viscosity of CRD-affected mucus to decrease to levels comparable to that of free NAC.

In sum, this data shows that the developed particles are capable of rapid mucolytic activity in a highly localized manner, allowing for the hypothesized rapid movement of these particles through CRD-affected mucus.

### 3.6. Evaluation of anti-inflammatory activity in 2D and lung-on-chip models

The anti-inflammatory activity of the dual-functional nanoparticles was evaluated using both conventional 2D cell culture systems and a physiologically relevant lung-on-chip model. The 2D culture assays enabled initial screening of nanoparticle-mediated cytokine suppression in a controlled inflammatory environment, while the organ-chip platform recapitulated key biomechanical and microenvironmental features of the lung, including air–liquid interface, directional flow, and cyclic stretch. This two-tiered approach allowed assessment of both the intrinsic biological activity of the formulation and its performance under more realistic airway conditions.

The secretion of pro-inflammatory cytokines such as IL-1β and TNFα by macrophages and other immune cells in the respiratory environment has been demonstrated to be a key part of repair and regeneration within the healthy lung. These cytokines have been shown to play a role in inducing the proliferation and differentiation of type II alveolar epithelial cells following injury, through the activation of multiple pathways, mainly NF-κB (Barnes, 2009; Katsura et al., 2019; O’Gorman et al., 2005; Yang et al., 2022). In addition, they have been implicated in airway repair through their induction of growth factors in mesenchymal stem cells and subsequent activation of the ERK1/2 pathway in lung epithelial cells (Broekman et al., 2016). In CRDs, their regenerative properties are subverted by their chronic presence within the respiratory tissues, prompting uncontrolled inflammation and epithelial dysfunction. This can include the goblet cell hyperplasia and upregulation of mucin gene expression that leads to mucus hypersecretion and hyperviscosity in conditions such as COPD (Rogers, 2007). This effect also contributes to the aberrant mucus production observed in CF, exacerbating the genetic component of the problem (Rogers, 2007). Considering this, both cytokines can be regarded as candidates for the induction of inflammation in a model of chronic airway inflammation. The choice of utilizing IL-1β to induce inflammation within this work was informed by the findings of authors such as Payne and colleagues, who found that IL-1β produced a stronger inflammatory response in A549 lung epithelial cells, which were also the model cell line of choice for the work described herein (Payne et al., 2019). This difference in inflammatory response was measured as a function of the production of two further key inflammatory cytokines, IL-6 and −8, by the A549 cells. Both IL-6 and −8 have been heavily implicated in airway inflammation seen in COPD, CF, IPF and asthma (El-Shimy et al., 2014; Le et al., 2014; Ordonez et al., 2000; Papiris et al., 2018; Rincon and Irvin, 2012).

**Fig. 6.**
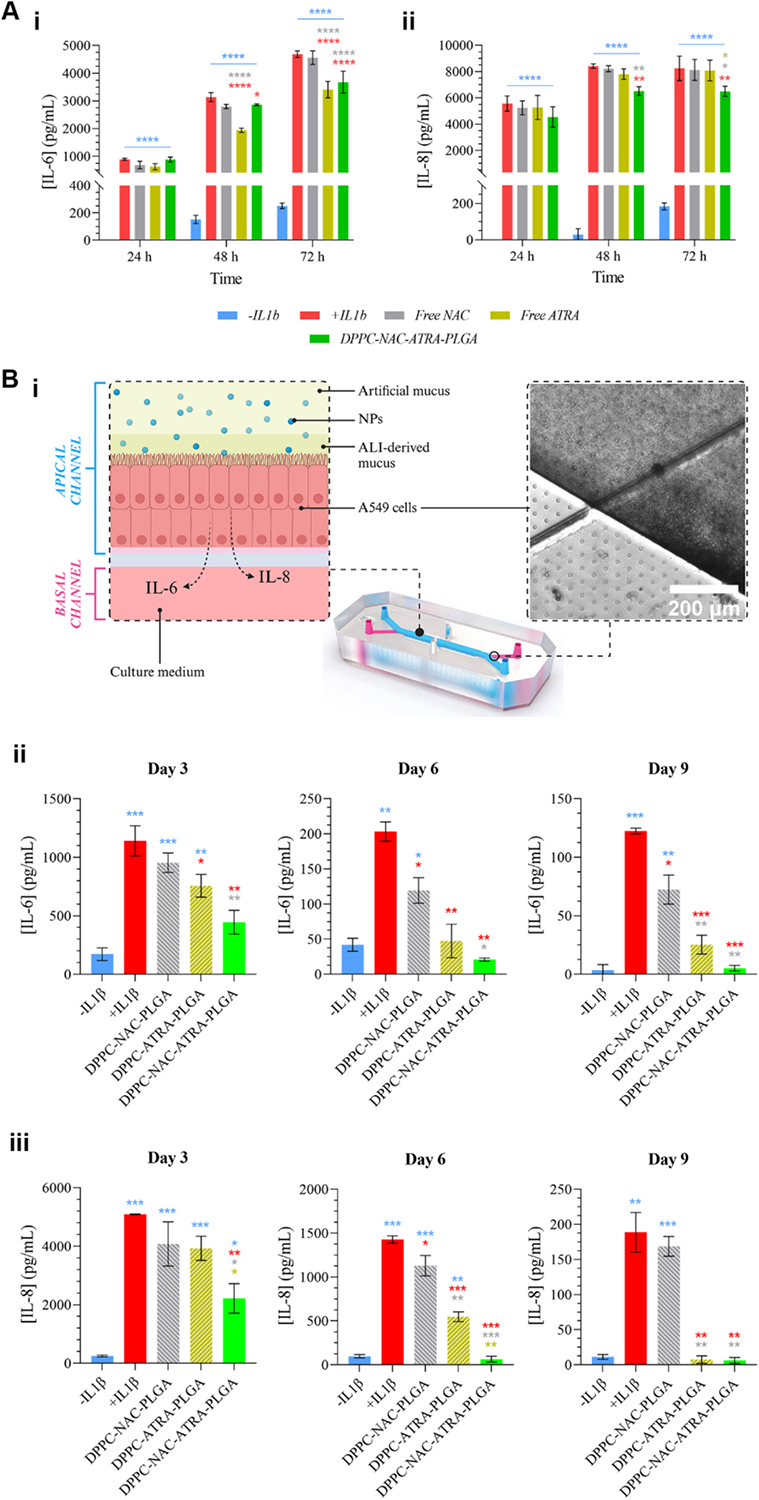
Evaluation of anti-inflammatory activity of dual-functional nanoparticles in conventional 2D cultures and a lung-on-chip model. **(A)** Exploratory 2D culture studies using inflamed A549 cells evaluating the potency of the final formulation and its components *via* quantification of **(i)** IL-6 and **(ii)** IL-8 secretion at 48 h and 72 h (*n* = 4). **(B)** Organ-on-chip experiments assessed nanoparticle performance under physiologically relevant conditions (air–liquid interface, flow, and cyclic stretch; *n* = 3) using a multi-dose regimen over 9 days. **(i)** Schematic of chip setup and representative micrograph. **(ii–iii)** Quantification of IL-6 and IL-8 levels in chip effluent. (*P ≤ 0.05, **P ≤ 0.01, ***P ≤ 0.001, ****P ≤ 0.0001.).

Initial work was carried out *via* standard 2D *in vitro* cell culture, examining the effects of the final formulation and its components. The concentration and duration of IL-1β exposure for all *in vitro* work was determined through exploratory studies performed through measurement of induced IL-6 and −8 levels as well as cytotoxicity in A549 cells (Fig. S5). The results of this work (presented in Fig. 6A) showed that a single dose of NPs at 1 mg/mL could induce a statistically significant reduction in levels of IL-6 after just 48 h (compared to diseased controls), albeit not approaching levels seen in healthy controls (Fig. 6A). This effect was maintained and enhanced at the 72 h timepoint. Free ATRA (added at a concentration matching the amount released by the NPs) also outperformed the nanoencapsulated ATRA at 48 h and beyond, which is to be expected given the release profile established previously. By contrast, preliminary studies assessing levels of IL-8 (Fig. 6A-ii) showed an intriguing trend: levels of IL-8 were not reduced by free ATRA and were only subject to reduction following 48 h incubation with ATRA-encapsulating NPs, a trend maintained at 72 h. This may be due to the higher aqueous dispersibility and uptake of ATRA in its nanoencapsulated form leading to higher cell exposure: Pu and coworkers demonstrated that higher concentrations of ATRA were required to reduce levels of IL-8 as compared to IL-6 (Pu et al., 2022). Their data showed that a 60 μM dose of ATRA on IPEC-J2 cells could reduce levels of IL-6 by approximately 4-fold, while IL-8 was only reduced by 2-fold, with further dose increases causing further reductions of IL-6 but none in IL-8.

Having established the short-term efficacy of the formulation in 2D culture, nanoparticle performance was next evaluated under more physiologically relevant conditions using a multi-dose regimen over an extended timescale. This work was conducted on Emulate Inc.’s lung-on-a-chip platform, which supports air–liquid interface (ALI) culture of model A549 cells in the presence of a flow-compatible artificial mucus (see Table S3 for its rheological profile). Under ALI conditions, A549 cells predominantly exhibit an alveolar epithelial phenotype, with subpopulations expressing markers of both alveolar type II (SP-C, TTF-1) and alveolar type I cells (AQP-5), alongside enhanced epithelial barrier formation (ZO-1) (Öhlinger et al., 2019; Wu et al., 2018). While ALI culture also induces mucin expression and apical mucus accumulation (*e.g*., MUC5B), canonical basal or goblet cell differentiation has not been reported for this model (Wu et al., 2018). Organ chips such as this have received increasing attention for their ability to replicate critical *in vivo* parameters, particularly flow and mechanical forces (Bennet et al., 2021; Leung et al., 2022; Zamprogno et al., 2021). They enable therapeutic response modelling without reliance on variable and cumbersome animal studies, and have been recognized by the USFDA through inclusion in its Innovative Science and Technology Approaches for New Drugs (ISTAND) Pilot Program (FDA, 2024). Besides the more physiologically relevant data obtained from such an experiment, we envisioned that, while inclusion of an ATRA-free control formulation would ensure accurate gauging of the anti-inflammatory effect of our formulation, inclusion of an NAC-free formulation would allow for further validation of the formulation’s mucolytic activity.

We hypothesized that DPPC-NAC-ATRA-PLGA would be capable of stronger and more significant reduction of inflammation than DPPC-ATRA-PLGA, at least at the early timepoints. Based on the results presented in Fig. 5, we expected that localized release of NAC around the individual NPs by DPPC-NAC-ATRA-PLGA would allow for the core to reach the inflamed epithelium more rapidly (and in turn, effectuate reduction of inflammation) than in the case of DPPC-ATRA-PLGA. The NAC-free formulation would have to rely solely on its ability to avoid charge interactions with mucin chains to move through the mesh.

Thus, we treated inflamed A549 cells with DPPC-NAC-PLGA, DPPC-ATRA-PLGA and our final DPPC-NAC-ATRA-PLGA formulation at concentrations of 2 mg/mL each in a supplementary mucus following establishment of native mucus production after ALI. This supplementary mucus was introduced through the apical channel of the organ chip as shown in Fig. 6B–i as the CF mucus model was too viscous for microfluidic flow. Cell culture media was not used for the introduction of treatment groups as it would not fully replicate the viscous nature of the contents of small airways affected by a CRD and would provide no additional resistance to NP diffusion. Chips were dosed every 72 h for a total of 9 days’ culture, and sampling of effluent media flowed through the basal channel of the chip was carried out immediately prior to each dosing. The 3-day dosing regimen was selected to match those frequently used in *in vivo* studies (Garcia et al., 2014; Xu et al., 2014). The results of this work are presented in Fig. 6B-ii/-iii. The DPPC-NAC-ATRA-PLGA group showed an ever-increasing propensity for anti-inflammatory activity through all tested timepoints, with trends in levels of IL-8 (Fig. 6B-iii) broadly followed those seen with IL-6, highlighting the superiority of the NAC + ATRA combination in allowing a much more rapid anti-inflammatory effect compared to NAC-free NPs.

An additional observation was the apparent anti-inflammatory activity of NAC, particularly its effect on IL-6 levels. This was not entirely unexpected: multiple other groups have shown a similar anti-inflammatory effect (Park et al., 2015; Santus et al., 2024; Wang et al., 2020). Montero and colleagues, for instance, noted the ability of chronic administration of low-dose NAC to reduce IL-6 production in polyinosinic-polycytidylic acid (Poly (I:C))-treated A549 cells (Montero et al., 2023). This effect is not limited to the *in vitro* space, with *in-* or *ex vivo* reports of this phenomenon published in extant literature (Askari et al., 2020; Calzetta et al., 2018; Saddadi et al., 2014). Saddadi *et al*. serve as one such example, having noted a significant decrease in serum IL-6 levels in patients receiving oral NAC while on haemodialysis (Saddadi et al., 2014). NAC’s effect on IL-8 is minimal, with statistically significant reductions relative to positive controls seen only on day 6. While NAC has been known to downregulate IL-8 levels, there is a complex interplay between times and dosing that underlies its anti-inflammatory activity as noted above, and it is entirely possible that the parameters of dosing for this formulation may need to be further fine-tuned to maximize the combinatorial effect of NAC and ATRA (Montero et al., 2023; Sadowska et al., 2007).

A gradual decline in overall IL-6 and IL-8 levels was observed over time (Fig. 6B-ii/iii). This can be attributed to cell sloughing under the shear forces generated by the high flow rates required to move even the less viscous synthetic mucus through the apical channel of the chips. To counter this, cells were first grown until several layers had formed. It is also possible that some cell death was experienced at extended dosing times because of ATRA’s anti-cancer activity against A549 cells. Nevertheless, the relative trends of cytokine levels hold throughout the experimental period.

### 3.7. Study limitations and translational considerations

Although the present work demonstrates that DPPC-PLGA nanoparticles can modulate the rheological properties of hyperviscous airway mucus and attenuate epithelial inflammatory responses *in vitro* and on a lung-on-chip model, several limitations should be noted when considering translation. From a materials standpoint, we used spherical NPs with relatively rigid polymer cores and did not systematically vary particle geometry or elasticity; as such, our findings speak specifically to the roles of hydrodynamic size and DPPC-mediated surface properties, while potential contributions of non-spherical shapes or deformable cores to airway mucus penetration remain to be explored in future work.

In addition, while the 7-day stability study in physiologic media showed that the near-neutral, DPPC-coated formulation maintains nanoscale size and colloidal integrity over experimentally relevant timescales, long-term storage is envisaged in the lyophilized state. All physicochemical characterization, mucus penetration, uptake, and lung-chip experiments were carried out on nanoparticles that had been lyophilized and reconstituted immediately prior to use, rather than on never-lyophilized suspensions, indicating that a single freeze-dry-–reconstitute cycle under our conditions does not produce gross aggregation or obvious lipid shell degradation. However, we did not undertake a formal long-term stability study of the lyophilized product under real-time or accelerated storage conditions. Detailed assessment of dry-state morphology, lipid phase behavior, and drug retention over extended storage will therefore be required as part of any future development program.

With respect to inhalation delivery, the data supports the feasibility of administering these nanoparticles as freshly reconstituted suspensions, given their colloidal stability assessed by us for up to 7 days, and timed NAC release in physiologic media. For aerosolization, we envisage delivery *via* a vibrating-mesh nebulizer in order to minimize shear stress on the DPPC-PLGA architecture. Our group has recently shown that Infasurf^®^ lung-surfactant–coated PLGA nanoparticles, which share a similar PLGA-core/DPPC-rich shell architecture, retain their hydrodynamic size, PDI, ζ, and morphology after passage through a commercial Aeroneb^®^ vibrating-mesh nebulizer, and that nebulization does not significantly alter their *in vitro* performance (Gonsalves and Menon, 2024). These findings, together with reports on other nebulized nanosuspensions, support the view that appropriately formulated hybrid nanoparticles can withstand mesh nebulization without substantial physicochemical disruption. Nevertheless, future studies will need to explicitly compare size, PDI, zeta potential, and dual-drug release profiles before and after nebulization, as well as to characterize aerosol performance using next generation impactor experiments and to map deposition patterns across the conducting airways and distal lung.

Finally, the present work did not evaluate *in vivo* biodistribution, clearance, or efficacy, nor did it address the consequences of repeated *in vivo* dosing over longer timeframes, which are particularly relevant for chronic respiratory diseases. Future *in vivo* studies should examine nanoparticle fate, mucus rheology, and cytokine panels in bronchoalveolar lavage fluid and airway tissue in appropriate disease models, building on the acute and sub-chronic responses observed here *in vitro*. Collectively, these limitations delineate the scope of the current study as a mechanistic and translational feasibility investigation, and they outline a clear path for subsequent optimization and preclinical development of this platform.

## 4. Conclusion

Airway inflammation is a significant driving force underlying multiple chronic respiratory diseases, contributing to pulmonary remodelling, declining quality of life and exercise capability, recurrent infection and exacerbation of the disease state overall. Current pharmaceutical approaches to address this issue rely on the separate administration of mucolytics and anti-inflammatory agents, the efficacy of which leaves much room for improvement. To address this, DPPC-coated PLGA nanoparticles were developed, encapsulating the mucolytic N-acetylcysteine in the lipid layer for rapid release and all-trans retinoic acid in the PLGA core for sustained release. The nanoparticles were of a size compatible with inhalation (<500 nm), were well tolerated by both epithelial and fibroblast cell lines, and demonstrated dose-dependent, non-saturable cellular uptake driven by their slightly positive zeta potential. The formulation induced significant reductions in both bulk and microrheological properties of hyperviscous diseased mucus, *via* decreased complex viscosity. When evaluated in a lung-on-chip model that mimics key airway biomechanical features, the dual-functional nanoparticles significantly reduced inflammatory cytokines IL-6 and IL-8 compared to mucolytic-free controls as early as 72 h after treatment, with effects maintained over a 9-day multi-dose regimen. These findings demonstrate that the combination of mucus penetration and dual-agent delivery can lead to rapid and sustained anti-inflammatory effects under physiologically relevant airway conditions. Moreover, this work underscores the value of lung-on-chip models for preclinical evaluation of inhaled nanotherapeutics in particular. Given the overlap between the platform’s relative simplicity and the robust effects it produces, it possesses real promise as an enhanced therapeutic approach for CRDs.

## Supplementary Material

1

## Figures and Tables

**Table 1:** Summary data from thermogravimetric analysis of NP formulation and components.

Sample	T_onset_ (°C)	T_max_ (°C)	Range (°C)	Residual mass (%)
*DPPC*	244.4	264.8	195.2–282.9	
	316.2	325.7	282.9–336.0	10.1
	330.7	337.8	330.7–493.4	
*PLGA NPs*	278.5	302.7	218.5–377.4	3.9
	419.1	422.2/438.7	377.4–493.4	
*DPPC-PLGA NPs*	242.6	261.6	178.7–280.5	
	309.0	318.5	280.5–327.2	9.1
	321.0	332.9	321.0–493.4	
*NAC*	171.5	195.0	115.0–259.3	20.0
	236.6	268.3	236.6–493.4	
*ATRA*	204.6	224.8	110.7–267.0	2.1
	281.5	297.3	267.0–493.4	
*DPPP-NAC-ATAA-PLGA NPs*	225.0	254.9	115.4–279.2	10.6
	317.5	325.5	279.2–493.4	

## Data Availability

Data will be made available on request.

## Appendix A. Supplementary data

Supplementary data to this article can be found online at https://doi.org/10.1016/j.ijpharm.2026.126688.
