# Supplementary material for: Mucopenetrative Lipid–Polymer nanoparticles show Potent Anti-Inflammatory activity in a human Lung-on-Chip model
Source: Int J Pharm. Author manuscript; Available in PMC 2026 Apr 30. (PMC13132617; doi:10.1016/j.ijpharm.2026.126688)
Supplement: 1 [file NIHMS2167814-supplement-1.docx]

*Supplementary Information for:*

**Mucopenetrative Lipid–Polymer Nanoparticles Show Potent Anti-Inflammatory Activity in a Human Lung-on-Chip Model**

Kalindu D. C. Perera, Alexandra K. Vasta and Jyothi U. Menon

**Table S1** Mathematical models and equations used for fitting *in vitro* release data of NAC and ATRA formulations.

| Model | Equation | Notes |
| --- | --- | --- |
| Higuchi | *Y=A_max_​⋅k_H_​⋅*$\surd t$ | NAC-only. |
| Burst + Higuchi | *Y=F_b​_+(A_max​_−F_b_​)k_H_*$\surd t$ | Used for NAC and ATRA nanoparticle datasets; A_max_​ fixed for ATRA, free for NAC. |
| Burst + First-order | *Y=F_b_​+(A_max_​−F_b_​)(1−e^−k^_1_^​t^)* | Used for ATRA nanoparticle release at both pH 7.4 and 6.5; A_max_ fixed to final value. Also used for NAC NPs. |
| Weibull | *Y=A_max_​[1−exp(−(*$\frac{t}{\alpha}$*​)^β^)]* | Explored for NAC. |


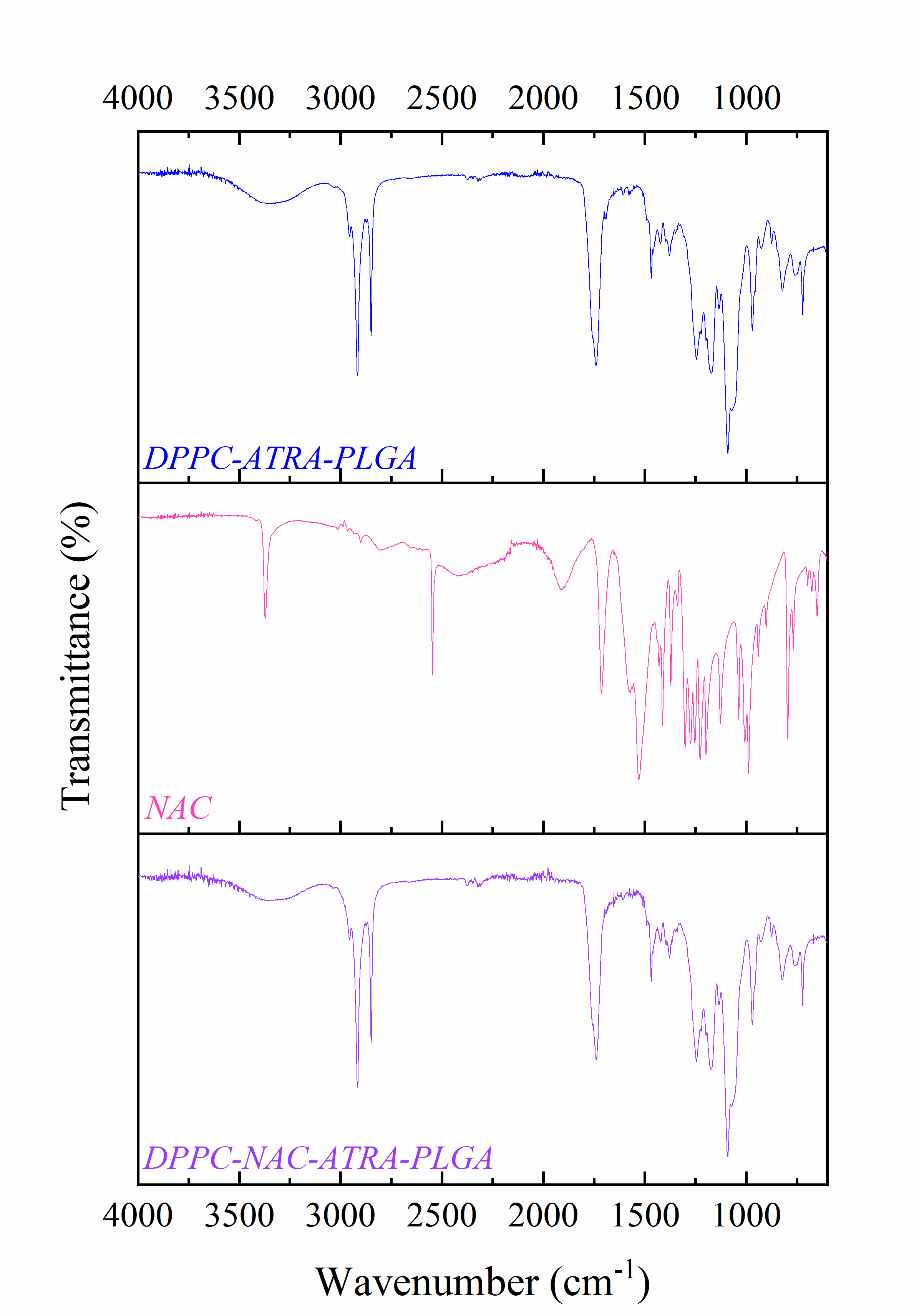


**Fig. S1** ATR-FTIR spectra tracking the encapsulation of N-acetylcysteine (NAC) within the DPPC coating on ATRA-encapsulated PLGA NPs. No clear sign of NAC’s incorporation within the lipid was visible.


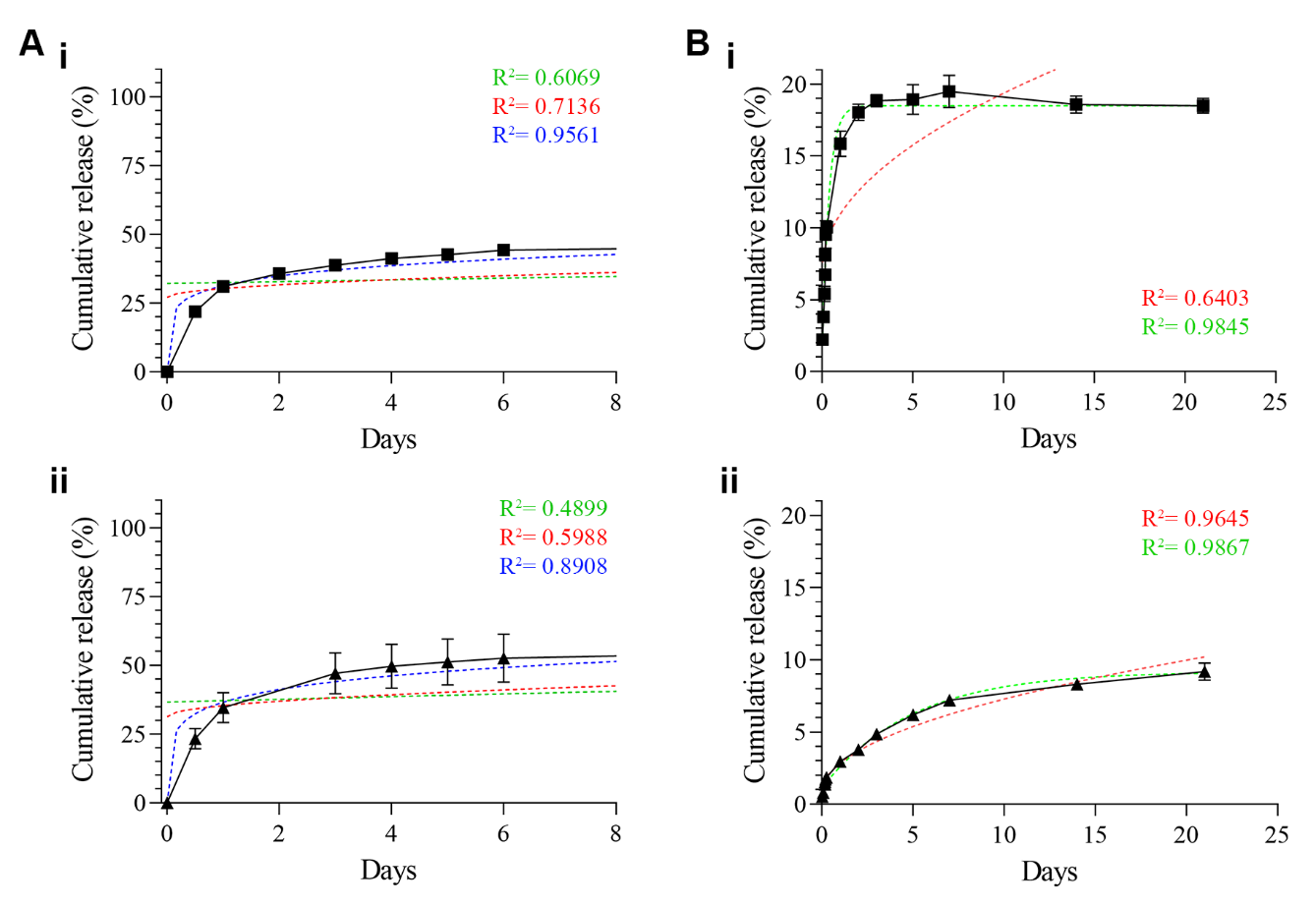


**Fig. S2** Nonlinear model fitting for **A)** NAC- and **B)** ATRA release profiles, with **i)** encapsulated drug at pH 7.4 and **ii)** encapsulated drug at pH 6.5. Dashed lines in red= Burst + Higuchi, green= Burst + First-order, blue= Weibull. Corresponding R² values for each model are shown within each panel.

**Table S2** Quantities of NAC and ATRA remaining within the DPPC- and PLGA matrices (per 5 mg of NPs) respectively at each time point tested.

| Time (days) | Amt. NAC remaining (µg) | | Amt. ATRA remaining (µg) | |
| --- | --- | --- | --- | --- |
|  | *pH 7.4* | *pH 6.5* | *pH 7.4* | *pH 6.5* |
| 0 | 540.1±12.2 | 493.4±11.2 | 215.5±9.8 | 185.0±6.4 |
| 0.021 (30 min.) | 422.1±5.2 | 378.4±18.2 | 210.7±0.4 | 184.0±0.1 |
| 0.042 (1 h) | 372.7±6.2 | 322.5±27.0 | 207.4±0.6 | 183.5±0.3 |
| 0.083 (2 h) | 347.3±8.4 | - | 203.9±1.1 | - |
| 0.125 (3 h) | 330.7±7.7 | 261.1±36.6 | 201.0±0.3 | 182.4±0.2 |
| 0.167 (4 h) | 317.7±7.6 | 248.6±39.3 | 198.0±1.0 | 182.1±0.1 |
| 0.208 (5 h) | 310.0±8.2 | 240.8±41.3 | 195.0±2.1 | 181.8±0.1 |
| 0.25 (6 h) | 301.4±7.9 | 234.2±43.0 | 194.0±0.6 | 181.6±0.3 |
| 1 | 277.9±10.6 | 147.2±91.3 | 181.3±1.9 | 179.5±0.2 |
| 2 | 262.8±14.9 | 145.2±52.9 | 176.7±1.2 | 178.0±0.1 |
| 3 | 248.8±16.9 | 152.6±57.7 | 174.9±0.9 | 176.0±0.0 |
| 5 | 215.2±27.5 | 148.0±49.1 | 174.7±2.2 | 173.5±0.3 |
| 7 | 186.5±33.6 | 136.3±55.9 | 173.5±2.4 | 171.6±0.3 |
| 14 | - | - | 175.5±1.3 | 169.6±0.3 |
| 21 | - | - | 176.5±1.6 | 168.0±1.1 |


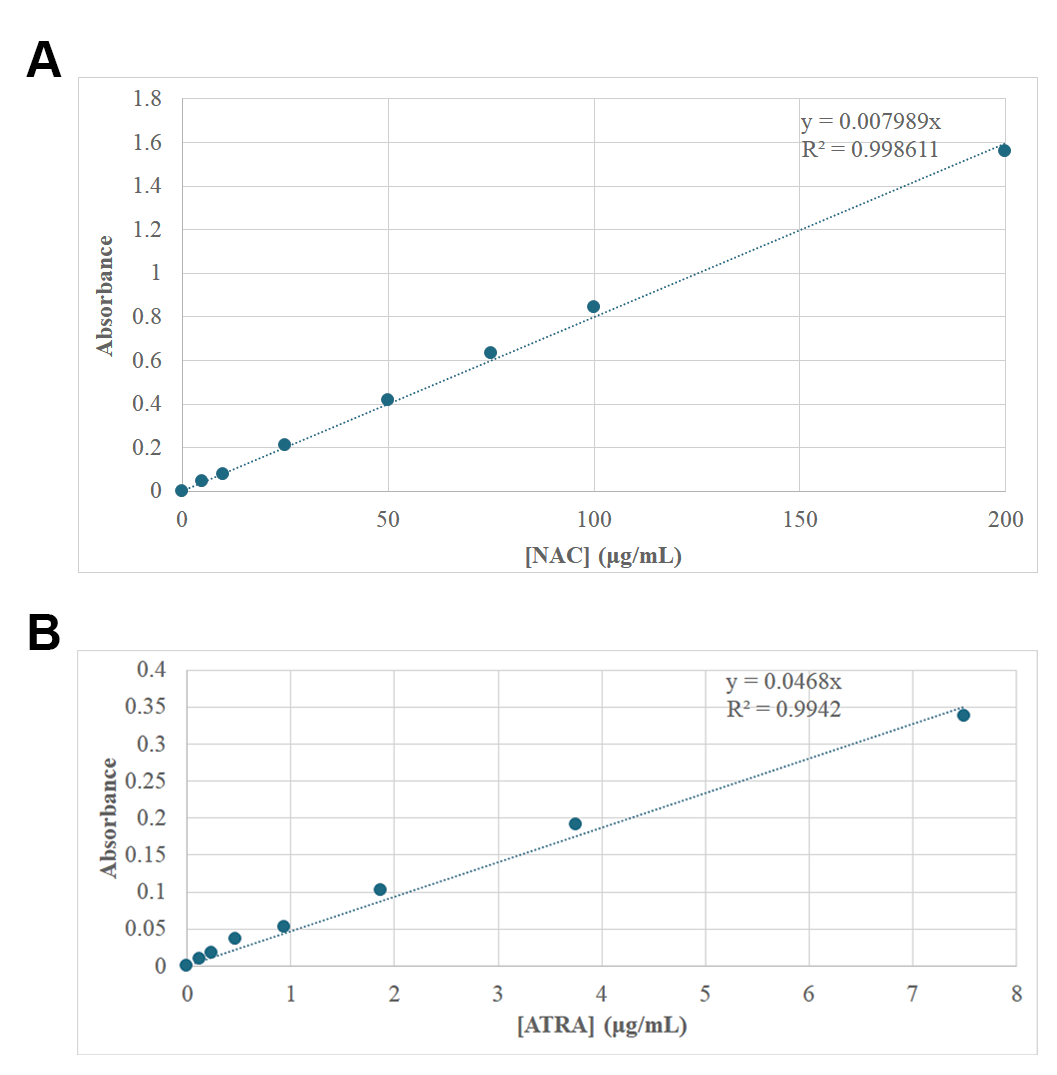


**Fig. S3** Calibration plots used for quantifying **A)** NAC and **B)** ATRA release *via* UV-vis spectrophotometry.


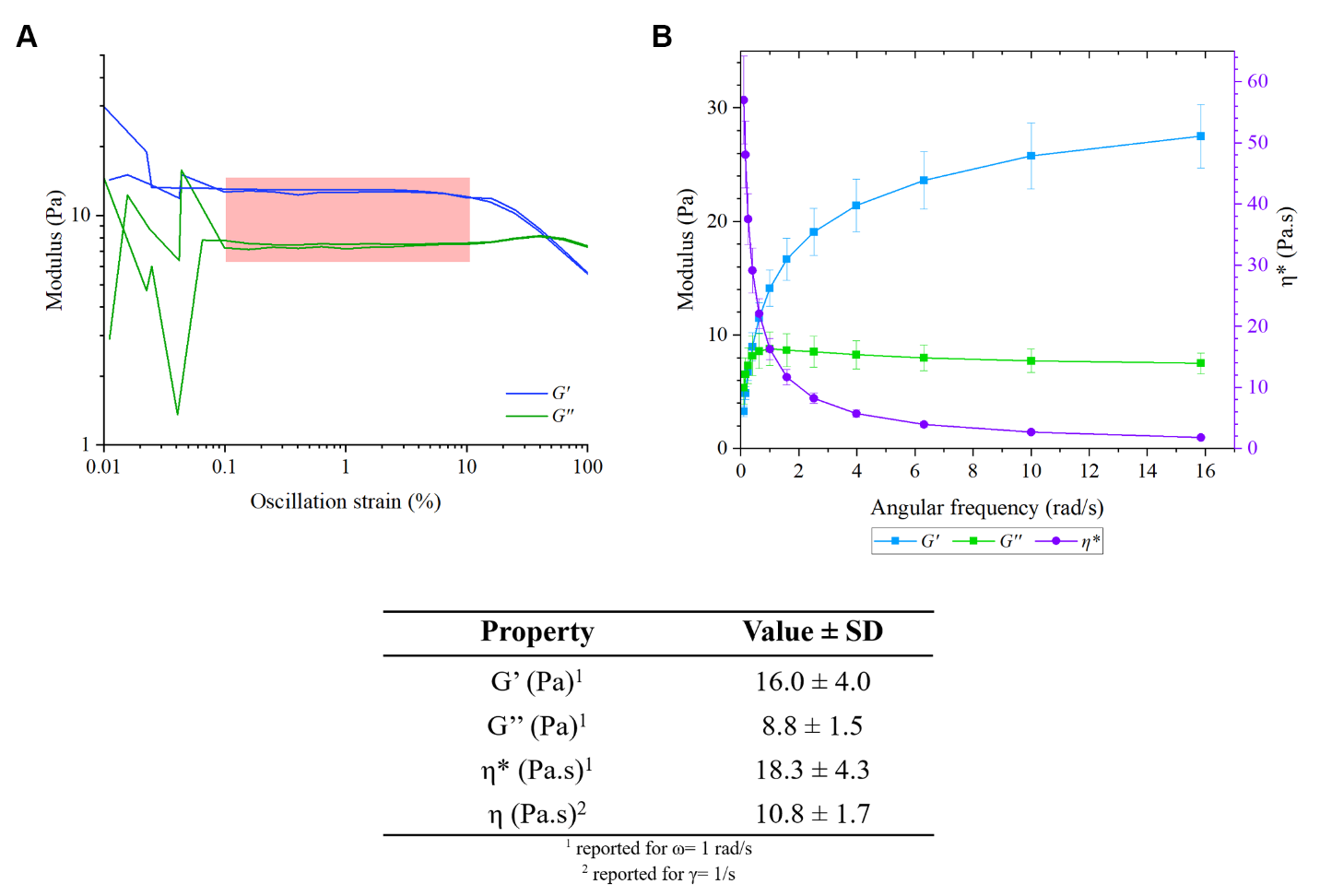


**Fig. S4** Rheological profile of the diseased *in vitro* model of mucus used in multiple studies, including identification of LVR, trends in G’, G’’ and η*, as well as summary data table (*n*=3).


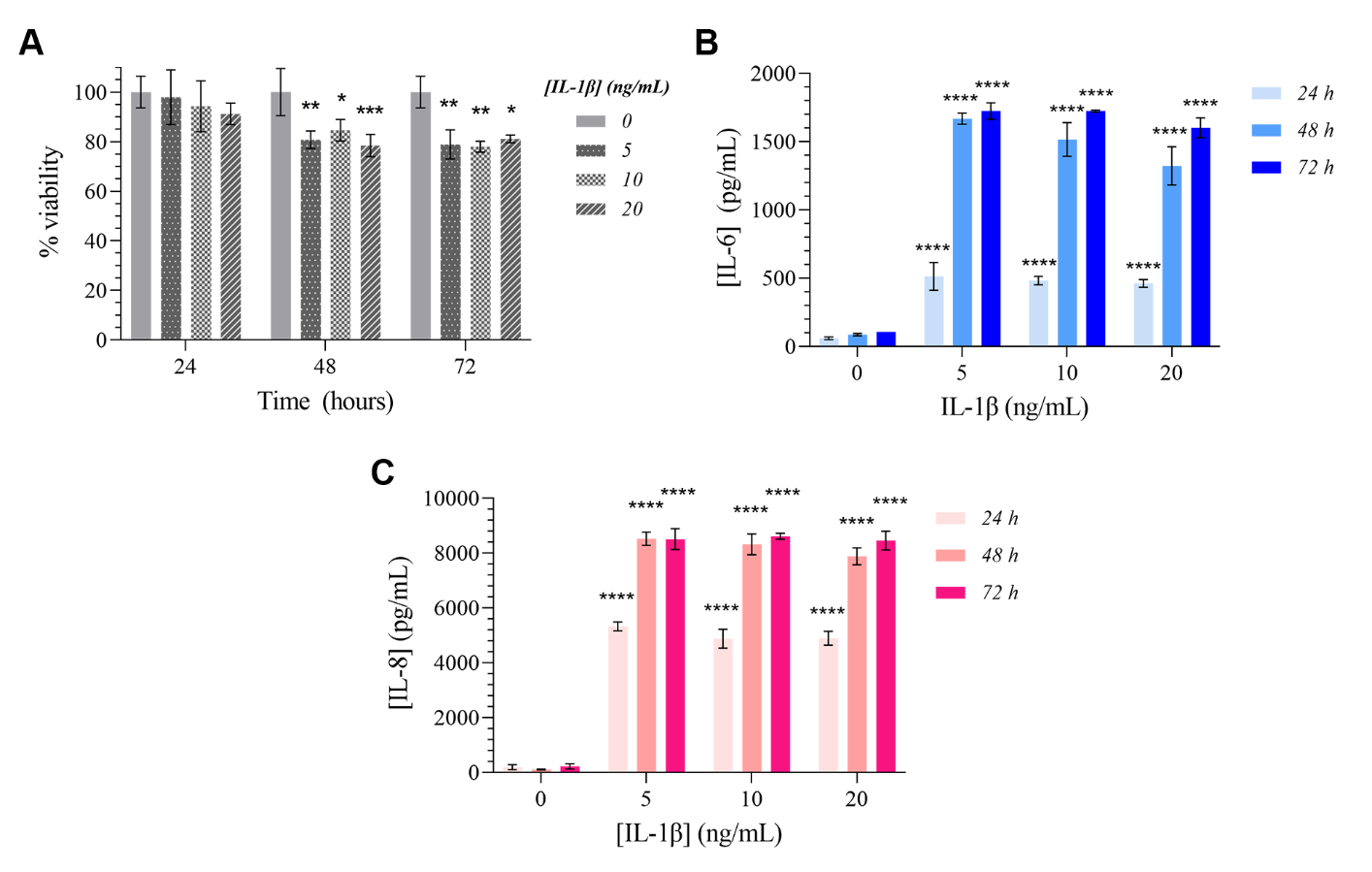


**Fig. S5** Basic data for identifying an optimal concentration and duration of exposure of IL-1β to inflame A549s, encompassing **A)** cytotoxicity study *via* MTT assay (significance indicated with respect to 0 ng/mL control at each timepoint), **B)** quantification of IL-6 and **C)** IL-8 levels in culture supernatant *via* ELISA (significance indicated with respect to 0 ng/mL control of each timepoint). (* P ≤ 0.05, ** P ≤ 0.01 *** P ≤ 0.001, **** P ≤ 0.0001)

**Table S3** Rheological profile of Biochemazone artificial mucus used for supplementing Emulate organ-on-chip work (*n*=3).

| Property | Value ± SD |
| --- | --- |
| G’ (Pa)^1^ | 0.727 ± 0.14 |
| G’’ (Pa)^1^ | 0.689 ± 0.09 |
| η* (Pa.s)^1^ | 1.00 ± 0.16 |
| η (Pa.s)^2^ | 0.673 ± 0.07 |

^1^ reported for ω= 1 rad/s

^2^ reported for γ= 1/s
